# Prevalence of Drug-Resistant Tuberculosis in Mainland China: Systematic Review and Meta-Analysis

**DOI:** 10.1371/journal.pone.0020343

**Published:** 2011-06-03

**Authors:** Yu Yang, Xiangwei Li, Feng Zhou, Qi Jin, Lei Gao

**Affiliations:** State Key Laboratory for Molecular Virology and Genetic Engineering, Institute of Pathogen Biology, Chinese Academy of Medical Sciences and Peking Union Medical College, Beijing, China; Swiss Tropical and Public Health Institute, Switzerland

## Abstract

**Background:**

The spread of drug-resistant tuberculosis (TB) is one of the major public health problems in the world. Surveillance of anti-TB drug resistance is important for monitoring TB control strategies. However, the status of drug-resistant TB in China has been reported inconsistently.

**Methods:**

We systematically reviewed published studies on drug-resistant TB in China until March 31, 2011, and quantitatively summarized prevalence and patterns of anti-TB drug resistance among new cases and previously treated cases, respectively.

**Results:**

Ninety-five eligible articles, published during 1993–2011, were included in this review. The meta-analyses showed that the prevalence of drug-resistant TB in new cases was 27.9% (95% CI, 25.6%–30.2%) (n/N = 27360/104356) and in previously treated cases was 60.3% (95% CI, 56.2%–64.2%) (n/N = 30350/45858). Furthermore, in these two study populations, the prevalence of multiple drug resistance was found to be 5.3% (95% CI, 4.4%–6.4%) (n/N = 8810/101718) and 27.4% (95% CI, 24.1%–30.9%) (n/N = 10486/44530) respectively. However, the results were found to be frequently heterogeneous (p for Q tests <0.001). The most common resistance was observed for isoniazid among both study populations. Different patterns of drug resistance were observed in the subgroup analysis with respect to geographic areas, drug susceptibility testing methods and subject enrollment time.

**Conclusions:**

Results of meta-analyses indicated a severe status of drug-resistant TB in China, which attaches an importance to strength TB prevention and control.

## Introduction

The prevalence of anti-tuberculosis (TB) drug resistance, which increases the rate of treatment failure and the costs of control, is a major challenge to global public health [1]–[3]. Together with delayed diagnosis and lack or inadequacy of TB control programs, the emergence of multidrug-resistant (MDR) TB has complicated the epidemiology of TB [4], [5]. It is estimated that, at present, 5% of the more than 9 million persons who develop TB around the world every year are infected with MDR-TB. China ranks second in terms of total numbers of MDR-TB cases which is only inferior to India [2], [6].

As reported, increased drug resistance rates to the first-line anti-TB drugs and MDR were observed in China in the past decade [7], [8]. However, these estimates presented high degrees of variability because the study setting were heterogeneous (i.e. population involved, geographic areas, outcome measurements, etc.). In 2000, the Fourth National Epidemiological Sampling Survey of Tuberculosis estimated the prevalence of TB infection in China is as high as 44.5%, and there were around 0.5 million drug-resistant TB cases among 200 million smear positive pulmonary TB patients [9]. Report of *National Baseline Survey of Drug-resistant Tuberculosis (2007–2008)* showed that resistance to at least one anti-TB drug (any resistance) among new cases with smear positive pulmonary TB was 35.2% (resistance to only one drug was 21.3% and to multidrug was 8.2%) and among previously treated cases was 55.2% (resistance to only one drug was 20.0% and to multidrug was 9.5%), respectively [10]. The high prevalence of drug-resistant TB suggested that persistent surveillance is essential and important to evaluate and improve the strategies of disease prevention and control.

The present study will be a priority to consider the prevalence and distribution of drug-resistant TB in mainland China using meta-analysis based on systematic review of articles published between February 1993 and March 2011. The relevant estimates were evaluated for new cases and previously treated cases, respectively, which will provide more detailed information to clearly overview the status of drug-resistant TB in China.

## Results

**Figure 1:**
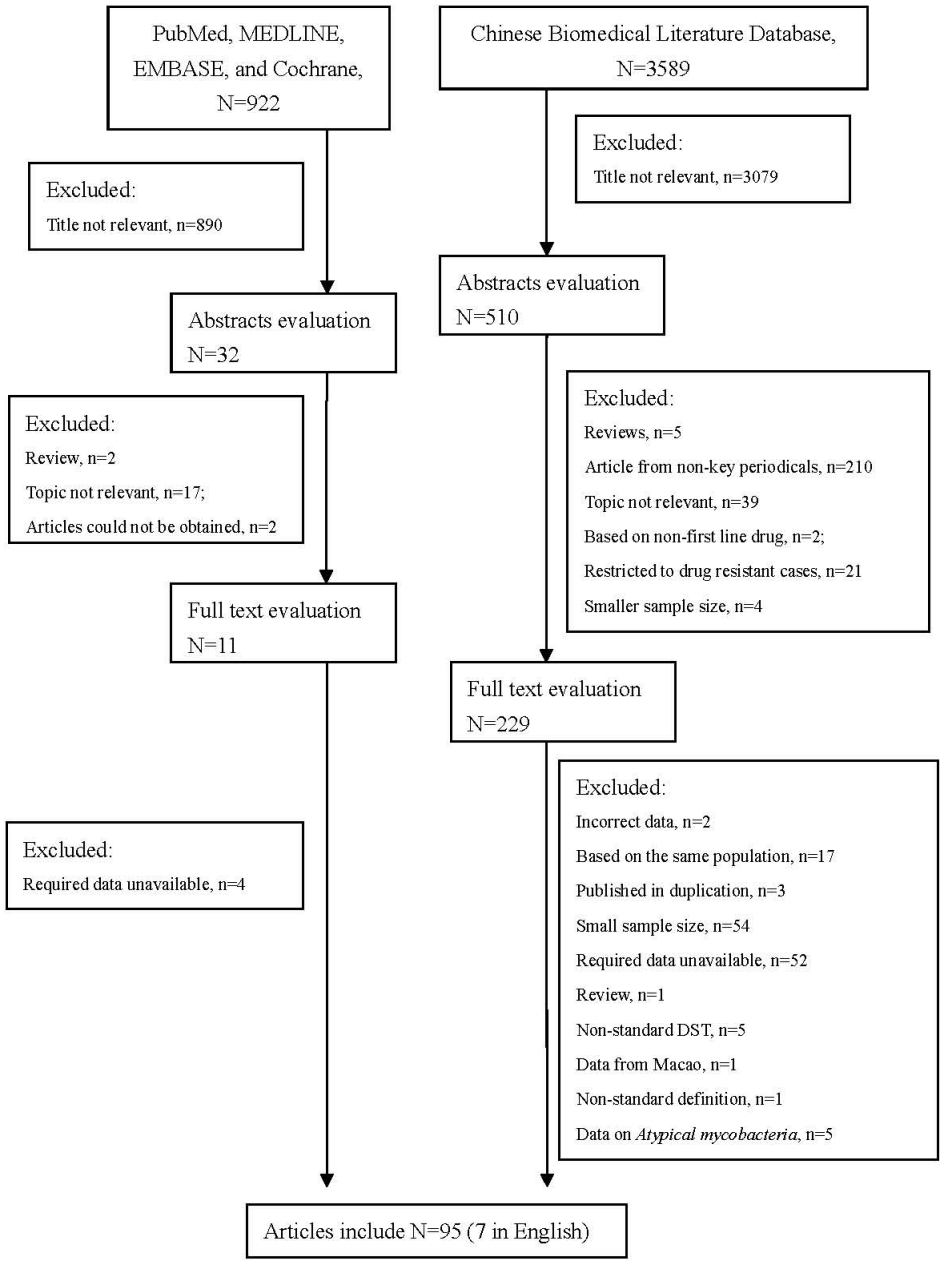
Flow diagram of study identification.

A total of 4511 articles were achieved by literature search using different combination of key terms from the databases as shown in **Figure 1**. After exclusion based on title and abstract evaluation, 240 articles were retrieved for detailed full-text evaluation. Finally, 95 studies, 7 in English and 88 in Chinese, addressing the prevalence of drug-resistant TB in new cases or in previously treated cases were identified, and please refer to **Table S1** for more detailed information on study identification.

As shown in **Table S2**, among the included articles, 85 provided information of new cases and 66 provided data of previously treated cases. 94 studies provided detailed results of drug susceptibility testing (DST) with respect to specific drugs. According to the methods of DST, 53, 33 and 9 studies used absolute concentration method, the proportion method and BACTEC, respectively. More studies were conducted in East (38) and South (15) China as compare to North (18), Central (14) and West (11) China.

**Table 1** shows the meta-analyses of the status of TB drug resistance in new cases in China. In total, 83 studies were included. The summarized prevalence of any drug resistance, mono-drug resistance and MDR were found to be 27.9% (25.6%–30.2%) (n/N = 27360/104356), 12.4% (11.1%–13.8%) (n/N = 5797/46049) and 5.3% (4.4%–6.4%) (n/N = 8810/101718), respectively. However, evident heterogeneity was observed (p<0.001). **Figure S1** shows forest plot of meta-analysis of any drug resistance. As shown in **Figure S2**, no evident publication bias was observed (p = 0.717 for Begg rank correlation analysis; p = 0.380 for Egger weighted regression analysis). In the stratified analyses, the prevalence of any drug resistance was observed to be varied by geographic areas (p<0.001) and DST methods (p<0.001). Lower rates were observed for studies from East and South China, and studies using the proportion method of DST. The end time of the cases enrollment (before 2000 or after 2000) did not significantly change the results (p = 0.916).

**Table 1:** Status of drug-resistant tuberculosis among new cases in China.

		Prevalence of Drug Resistance(95% CI) (%)	n/N	No. of Studies	Heterogeneity Test	
					I^2^(%)	p
**Any drug resistance**	**Total**	27.9 (25.6–30.2)	27360/104356	83	98.2	<0.001
	**Stratified by geographic areas**					
	North China	34.7(28.9–41.0)	12258/40676	17	98.8	<0.001
	East China	21.5(18.8–24.5)	6967/36337	36	97.3	<0.001
	South China	26.9(23.1–31.0)	3888/15968	11	96.0	<0.001
	Central China	36.4(30.2–43.1)	2751/7839	12	96.4	<0.001
	West China	30.3(22.9–38.8)	1786/5254	9	97.4	<0.001
	**Stratified by years**					
	Before (include) 2000	26.2(22.4–30.5)	6890/27874	36	97.8	<0.001
	After 2000	26.0(22.7–29.5)	9512/38426	38	98.1	<0.001
	**Stratified by DST methods**					
	Absolute concentration method	28.0(24.7–31.5)	15970/60180	45	98.3	<0.001
	The proportion method	26.7(23.4–30.2)	8965/32620	32	97.8	<0.001
	BACTEC	29.7(22.4–38.2)	1699/6709	7	97.6	<0.001
**Mono-drug resistance**	**Total**	12.4(11.1–13.8)	5797/46049	59	94.4	<0.001
	**Stratified by geographic areas**					
	North China	11.9(8.4–16.6)	1044/7152	8	96.5	<0.001
	East China	11.3(9.1–13.9)	1652/15440	23	95.0	<0.001
	South China	14.4(11.7–17.6)	2065/15968	11	95.3	<0.001
	Central China	13.1(10.2–16.6)	472/3637	10	85.6	<0.001
	West China	13.1(9.5–17.9)	380/2923	6	92.7	<0.001
	**Stratified by years**					
	Before (include) 2000	10.9(9.6–12.5)	2535/24073	31	91.3	<0.001
	After 2000	12.8(10.5–15.4)	1934/14521	21	94.7	<0.001
	**Stratified by DST methods**					
	Absolute concentration method	11.8(9.7–14.2)	1759/15607	28	94.2	<0.001
	The proportion method	13.4(11.6–15.3)	3351/23995	24	94.0	<0.001
	BACTEC	11.8(7.9–17.3)	687/6447	6	96.5	<0.001
**Multi-drug resistance**	**Total**	5.3(4.4–6.4)	8810/101718	76	98.2	<0.001
	**Stratified by geographic areas**					
	North China	6.6(4.5–9.5)	5582/38413	14	98.4	<0.001
	East China	3.8(3.1–4.6)	1244/35307	33	90.8	<0.001
	South China	4.8(3.6–6.4)	818/15968	11	92.8	<0.001
	Central China	10.1(7.2–14.2)	704/7725	11	95.0	<0.001
	West China	7.3(5.1–10.3)	556/6493	9	93.0	<0.001
	**Stratified by years**					
	Before (include) 2000	4.8(3.7–6.1)	1485/27969	35	95.4	<0.001
	After 2000	5.3(4.4–6.4)	2020/37354	35	94.3	<0.001
	**Stratified by DST methods**					
	Absolute concentration method	4.4(3.2–6.0)	5955/56026	38	98.2	<0.001
	The proportion method	6.1 (4.6–7.9)	2265/34136	32	97.4	<0.001
	BACTEC	7.4(5.2–10.3)	494/6709	7	92.5	<0.001

Abbreviation: BACTEC, use of BACTEC 460 TB system in tuberculosis diagnosis; DST, drug-susceptibility testing; n, number of events(drug resistance); N, total number of patients from the included studies.

As shown in **Table 2**, the status of TB drug resistance in previously treated cases in China was evaluated by meta-analysis. In total, 62 studies were included and their results were found to be heterogeneous in the meta-analyses as well (p<0.001). The summarized prevalence of any drug resistance, mono-drug resistance and MDR in the study population were found to be 60.3% (56.2%–64.2%) (n/N = 30350/45858), 15.6%(13.5%–18.0%) (n/N = 2462/15177) and 27.4% (24.1%–30.9%) (n/N = 10486/44530), respectively. Forest plot for the analysis of any drug resistance was shown in **Figure S3**. Some evidence for publication bias was observed (p = 0.951 for Begg rank correlation analysis; p = 0.002 for Egger weighted regression analysis), and please refer to **Figure S4** for funnel plot. In the subgroup analyses, the prevalence of any drug resistance was observed to be influenced by geographic areas (p<0.001), DST methods (p<0.001) and enrollment period (p<0.001). Lower rates were observed for studies from East and South China, studies using the proportion method of DST, and studies with the end time of subject enrollment after 2000.

**Table 2:** Status of drug-resistant tuberculosis among previously treated cases in China.

		Prevalence of Drug Resistance(95% CI) (%)	n/N	No. of Studies	Heterogeneity Test	
					I^2^(%)	p
**Any drug resistance**	**Total**	60.3 (56.2–64.2)	30350/45858	62	98.2	<0.001
	**Stratified by geographic areas**					
	North China	67.8(60.7–74.2)	18184/25463	14	98.4	<0.001
	East China	58.8(52.0–65.3)	9022/14362	27	98.2	<0.001
	South China	55.2(43.0–61.4)	1537/3023	9	95.8	<0.001
	Central China	61.1(52.6–69.1)	1575/2781	9	94.6	<0.001
	West China	66.3(47.5–81.1)	1848/2667	5	97.1	<0.001
	**Stratified by years**					
	Before (include) 2000	61.5(53.1–69.3)	5303/8537	21	97.8	<0.001
	After 2000	56.1(49.8–62.3)	6559/11856	33	97.5	<0.001
	**Stratified by DST methods**					
	Absolute concentration method	66.7(62.5–70.7)	23557/32794	29	97.3	<0.001
	The proportion method	53.2(49.2–57.2)	5755/10478	29	93.0	<0.001
	BACTEC	71.6(52.6–85.2)	2469/3644	5	98.9	<0.001
**Mono-drug resistance**	**Total**	15.6(13.5–18.0)	2462/15177	39	92.7	<0.001
	**Stratified by geographic areas**					
	North China	25.2(14.2–40.6)	916/5028	8	98.4	<0.001
	East China	14.6(12.1–17.6)	501/3346	15	79.8	<0.001
	South China	16.7(15.2–18.4)	475/2857	8	25.3	0.226
	Central China	12.8(10.2–15.9)	206/1605	6	63.4	0.018
	West China	11.6(6.7–19.4)	364/2341	3	83.1	0.003
	**Stratified by years**					
	Before (include) 2000	14.6(12.5–17.0)	660/4474	15	74.4	<0.001
	After 2000	14.2(12.4–16.3)	643/4386	16	68.9	<0.001
	**Stratified by DST methods**					
	Absolute concentration method	16.0(12.4–20.6)	642/3915	17	92.0	<0.001
	The proportion method	16.3(12.5–21.5)	1236/7618	18	95.7	<0.001
	BACTEC	15.8(13.6–18.3)	584/3644	5	63.9	0.026
**Multi-drug resistance**	**Total**	27.4(24.1–30.9)	10486/44530	57	97.9	<0.001
	**Stratified by geographic areas**					
	North China	25.9(20.3–32.4)	4760/24648	11	98.2	<0.001
	East China	26.4(21.4–32.1)	3274/13448	25	97.4	<0.001
	South China	21.5(16.2–27.9)	672/3023	9	93.1	<0.001
	Central China	29.2(24.7–34.1)	798/2660	8	85.4	<0.001
	West China	38.2(23.8–55.0)	1380/3189	6	97.9	<0.001
	**Stratified by years**					
	Before (include) 2000	25.9(20.7–31.9)	2097/8403	20	96.6	<0.001
	After 2000	25.2(20.8–30.3)	2955/11462	30	96.8	<0.001
	**Stratified by DST methods**					
	Absolute concentration method	24.7(21.0–28.9)	6055/31058	24	97.4	<0.001
	The proportion method	27.7(23.6–32.2)	3290/10886	29	95.5	<0.001
	BACTEC	39.8(27.2–53.9)	1405/3644	5	98.0	<0.001

Abbreviation: BACTEC, BACTEC 460 TB system of anti-tubercular screening; DST, drug-susceptibility testing; n, number of events(drug resistance); N, total number of patients from the included studies.

**Table S3** shows the meta-analyses on patterns of TB drug resistance in new cases in China. The most common drug resistance was observed for isoniazid and streptomycin with a summarized any prevalence of 15.3% (13.1%–17.7%) and 14.3% (12.4%–16.5%) respectively. Among MDR, the most common form of drug resistance was HRS with a prevalence of 2.0% (1.5%–2.5%). Distribution of anti-TB drug resistance in previously treated cases was estimated by means of meta-analysis as shown in **Table S4**. Similarly, the most common resistance was observed for isoniazid and rifampicin with a summarized combined prevalence of 41.5% (36.6%–46.6%) and 34.6% (30.1%–39.4%) respectively.

In additional sensitivity analyses, excluding studies with smaller sample size, the prevalence of any drug resistance was not substantially changed in new cases as 27.7% (25.0%–30.5%) (p = 0.603) but statistically increased in previously treated cases as 63.7% (59.1%–68.0%) (p<0.001).

## Discussion

This review addressed the prevalence and distribution of drug-resistant TB in mainland China. Ninety-five articles performed DST among new cases or previously treated cases were identified for meta-analysis. The results were found to be frequently heterogeneous. Our analyses showed that the prevalence of drug-resistant TB in new cases was 27.9% (25.6%–30.2%) (n/N = 27360/104356) and in previously treated cases was 60.3% (56.2%–64.2%) (n/N = 30350/45858). Furthermore, in these two study populations, the prevalence of MDR was found to be 5.3% (4.4%–6.4%) (n/N = 8810/101718) and 27.4% (24.1%–30.9%) (n/N = 10486/44530), respectively. Different patterns of drug resistance were observed with respect to geographic areas, study period and methods of drug-susceptibility testing.

Streptomycin, the first anti-TB drug, was introduced in 1944 and showed impressive therapeutic outcomes [11]. However, monotherapy was found to be ineffective to against TB because of the easily appeared drug resistance. Combination therapy then was developed and widely used which can reach better anti-TB effect and largely reduce the rate of resistance to multiple drugs. Thereafter, drug-resistant TB was recognized as a major problem again since MDR-TB was reported in worldwide. Multiple explanations have been suggested to contribute to the spread of MDR-TB around the world, including lack or inadequacy of anti-TB strategies, inadequate treatment programs, and insufficient resources, as well as delayed diagnosis of TB [5], [12]. In China, the spread of drug-resistant TB draws attention since the Fourth National Epidemiological Sampling Survey of Tuberculosis in 2000. Therefore, in 2002, *Guideline on Enforcement of Chinese Tuberculosis Control Program* was published by the Department of Disease Control of Ministry of Health [7], [13]. This Guideline was constituted to standardize programs for TB prevention and treatment, health workers will directly observe all patients to help improve TB case management and to enhance drug resistance surveillance and control.

In our analyses, TB patients were divided into two subgroups: new cases and previously treated cases. The prevalence of the drug resistance among new cases will reflect the transmission status of drug-resistant TB among general population. We found more than a quarter of the newly diagnosed TB cases (27.9%) were resistant to at least one drug and 5.3% of them were MDR. Such a high prevalence is a big challenge to achieve more than 90% cure rates [14]–[16]. The prevalence of the drug resistance among previously treated cases might partly reflect the treatment efficacy. Drug resistance was found might explain more than a half of re-treatments (60.3%), which suggests previously treated cases should be a major target population for drug resistance surveillance. Also, standard treatment strategies and availability of second-line drugs for drug-resistant TB should be paid attention to. In the other hand, there was still a proportion of re-treatment was not related to drug resistance, it indicates cases management should be improved to minimize the drop-out and lost to follow up [17].

Stratified analyses were performed in the present study according to the geographic areas, enrollment time and DST methods. We found most of the included studies came from East China and South China and relatively lower drug resistant rates were reported by them. The better social-economic status might contribute to such an observation because of the corresponding higher medical and public health levels of these two areas. Potential bias caused by the limited number of studies from areas with lower social-economic status could not be excluded. To minimize the gap between the areas is also an important project to the health system [18]. We found DST methods also influence the drug resistance rates. It might be explained by the different sensitivity of the methods and reminds us to consider this important issue when systematically reviewing the results of different studies. In addition, a decline of drug resistance was observed, especially among previously treated cases, with respect to the end of enrollment time. It is imagined that standard surveillance and treatment programs applied after 2000 might played a role in reducing drug resistance. Due to more detailed data on specific time point could not be obtained from the included studies, it is difficult to compare our analyses with the results of national survey performed during 2007–2008 [10]. However, an important point should be noticed that the prevalence of MDR was as low as 9.5% among previously treated cases as reported by the national survey. But it is much higher in our meta-analyses performed among all included studies (27.4%) and studies enrolled subjects after 2000 (25.2%). The characteristics of the studied previously treated cases, like the proportions of treatment failure cases and treatment discontinued cases, might be one of the major factors contribute to such an inconsistency. Our sensitivity analyses also indicated the result of previously treated cases was much sensitive as compared to the estimate of new cases. Therefore, the status of drug-resistant TB among previously treated cases should be assessed in more detail according to the characteristics of study population in the future studies.

Some limitations of this systematic review should be considered for results interpretation. First, the potential influence of age, sex, ethnicity, economic level and life styles could not be analyzed due to the limited information obtained from the original articles. Second, potential publication bias cannot be completely excluded as pronounced results are more likely to be published, especially indication of some publication bias was observed in our analyses. Third, most included studies were hospital-based rather than population-based which makes the results more prone to potential selection bias. Fourth, potential language bias could not be excluded completely because our literature search only considered articles published in English and in Chinese. Fifth, evident heterogeneity observed in included studies should be kept in mind when interpret our results [19]. As suggested by our subgroup analyses, it might be explained, at least in part, by various study populations and study design (e.g. study period and DST methods).

In conclusion, this systematic review summarized the prevalence and distribution of drug-resistant TB among new cases and previously treated cases in mainland China. Our results suggested that effective strategies to minimize the acquired drug resistance, to control the transmission of resistance, to improve the diagnosis measures should be attached importance for TB control in China.

## Materials and Methods

### Literature identification

Studies addressing drug resistance of TB in mainland China, published in English or Chinese, were identified by searching for original articles in several electronic databases until 31 March 2011. Chinese BioMedical Literature Database (1978-), PubMed (1946-), MEDLINE (1947-), EMBASE (1974-) and the Cochrane CENTRAL database (1972-). Combinations of the key words “tuberculosis”, “drug resistance” and “China” were used to screen for potentially relevant studies. Additional studies were also indentified by cross-referencing.

### Inclusion and Exclusion criteria

Original articles presented cross-sectional or cohort studies and reported the prevalence of drug resistance of TB in mainland China were considered. The included studies should provide drug resistance data of either new cases or previously treated cases, or both. If the study was reported in duplicate, the version firstly published or published in English was included. Review articles, congress abstracts, studies reported in languages other than English or Chinese, data from the regions of China other than mainland (i.e. TaiWan, HongKong and Macao), and studies using non-standard study methods and definition were excluded. In order to minimize the potential bias caused by too small sample size, articles with less than 100 subjects in the subgroup of new cases and previously treated cases were excluded.

### Data extraction

For all studies, we extracted the following data from the original publications: first author and year of publication, study enroll time and population, distribution of age and sex in the study population, DST methods, prevalence of mono- and multi-drug resistance and any drug resistance. Data on most widely used first-line anti-TB drugs (i.e. H, isoniazid; R, rifampin; E,ethambutol; and S, streptomycin) were extracted as well. Different patterns of multi-drug resistance were accordingly indicated by HR, HRS, HRE and HRSE. DST methods include absolute concentration method, the proportion method and BACTEC method. The term “BACTEC” refer to use of BACTEC 460 TB system in the diagnosis of tuberculosis. The end time of subject enrollment was classified into before (include) 2000 and after 2000 considering the launch of “TB Treatment and Control Planning of China (2001–2010)” which strengthened TB prevention and control in countywide. Literature identification and data extraction was performed by two researchers independently. The discrepancies between the reviewers in either the decision on inclusion or exclusion of studies or on data extraction were discussed by the study team to make the final decision.

In the text, the term “resistance among new cases” refers to patients with pulmonary TB who have never received anti-TB drugs or have received less than one month of treatment. The term “resistance among previously treated cases” is used to refer to patients who had previously received anti-TB drugs for one month or more. “Mono resistance” was used to define the resistance to only one first-line anti-TB drug. “Multi-drug resistance” (MDR) was used to define the resistance to at least isoniazid and rifampin. “Any drug resistance” referred to resistance to any drug regardless of mono-resistance or MDR.

### Statistical analysis

Meta-analyses were carried out using Comprehensive Meta-Analysis (V2.0, Biostat, Englewood, NJ, USA) for the prevalence of drug-resistant TB among new cases and previously treated cases, respectively [20]. Stratified analyses were subsequently performed with respect to the geographic areas, the end time of the enrollment period and DST methods (absolute concentration method, the proportion method and BACTEC 460 TB system). Random effects models were used, taking into account the possibility of heterogeneity between studies, which was tested with the Q test and I^2^ test. Begg rank correlation and Egger weighted regression methods were used to statistically assess publication bias (p<0.05 was considered indicative of statistically significant publication bias). Differences of the results in the subgroup analyses were assessed by *chi-square* tests. Sensitivity analyses were performed after excluding those studies with sample size less than 200 for both new cases and previously treat cases.

## Supporting Information

Figure S1 **Forest plot of the meta-analysis on any drug resistance in new cases.** (“*” in the figure means studies reported by the same publication).
(TIF)

Figure S2 **Funnel plot of the meta-analysis on any drug resistance in new cases.**
(TIF)

Figure S3 **Forest plot of the meta-analysis on any drug resistance in previously treated cases.** (“*” in the figure means studies reported by the same publication).
(TIF)

Figure S4 **Funnel plot of the meta-analysis on any drug resistance in previously treated cases.**
(TIF)

Table S1 **Included and excluded articles after full-text evaluation.**
(DOC)

Table S2 **Detailed information of the included studies.**
(XLS)

Table S3 **Distribution of different patterns of TB drug resistance among new cases in China.**
(DOC)

Table S4 **Distribution of different patterns of TB drug resistance among previously treated cases in China.**
(DOC)

## References

[1] Chiang CY, Schaaf HS (2010). Management of drug-resistant tuberculosis.. Int J Tuberc Lung Dis.

[2] Loddenkemper R, Hauer B (2010). Drug-resistant tuberculosis: a worldwide epidemic poses a new challenge.. Dtsch Arztebl Int.

[3] Wright A, Zignol M, Van Deun A, Falzon D, Gerdes SR (2009). Epidemiology of antituberculosis drug resistance 2002–07: an updated analysis of the Global Project on Anti-Tuberculosis Drug Resistance Surveillance.. Lancet.

[4] Caminero JA (2010). Multidrug-resistant tuberculosis: epidemiology, risk factors and case finding.. Int J Tuberc Lung Dis.

[5] Zignol M, Hosseini MS, Wright A, Weezenbeek CL, Nunn P (2006). Global incidence of multidrug-resistant tuberculosis.. J Infect Dis.

[6] Organization WH (2008). Anti-tuberculosis drug resistance in the world..

[7] Shen X, DeRiemer K, Yuan ZA, Shen M, Xia Z (2009). Drug-resistant tuberculosis in Shanghai, China, 2000–2006: prevalence, trends and risk factors.. Int J Tuberc Lung Dis.

[8] Wang D, Yang C, Kuang T, Lei H, Meng X (2010). Prevalence of Multidrug and extensively drug-resistant tuberculosis in Beijing, china: A hospital-based retrospective study.. Japanese Journal of Infectious Diseases.

[9] Group NTS (2002). Report on fourth national epidemiological sampling survey for tuberculosis.. Chin J Tuberc Respir Dis.

[10] Chinese Ministry of Health (2010).

[11] Schatz A, Bugie E, Waksman SA (2005). Streptomycin, a substance exhibiting antibiotic activity against gram-positive and gram-negative bacteria. 1944.. Clin Orthop Relat Res.

[12] Bammann RH, Zamarioli LA, Pinto VS, Vazquez CM, Litvoc MN (2010). High prevalence of drug-resistant tuberculosis and other mycobacteria among HIV-infected patients in Brazil: a systematic review.. Mem Inst Oswaldo Cruz.

[13] Hu D, Liu X, Chen J, Wang Y, Wang T (2008). Direct observation and adherence to tuberculosis treatment in Chongqing, China: a descriptive study.. Health Policy Plan.

[14] Xianyi C, Fengzeng Z, Hongjin D, Liya W, Lixia W (2002). The DOTS strategy in China: results and lessons after 10 years.. Bull World Health Organ.

[15] Xu B, Dong HJ, Zhao Q, Bogg L (2006). DOTS in China - removing barriers or moving barriers?. Health Policy Plan.

[16] Menzies D, Benedetti A, Paydar A, Royce S, Madhukar P (2009). Standardized treatment of active tuberculosis in patients with previous treatment and/or with mono-resistance to isoniazid: a systematic review and meta-analysis.. PLoS Med.

[17] Jassal MS, Bishai WR (2010). Epidemiology and challenges to the elimination of global tuberculosis.. Clin Infect Dis.

[18] Van Deun A, Martin A, Palomino JC (2010). Diagnosis of drug-resistant tuberculosis: reliability and rapidity of detection.. Int J Tuberc Lung Dis.

[19] Higgins JP, Thompson SG, Deeks JJ, Altman DG (2003). Measuring inconsistency in meta-analyses.. BMJ.

[20] Gao L, Tao Y, Zhang L, Jin Q (2010). Vitamin D receptor genetic polymorphisms and tuberculosis: updated systematic review and meta-analysis.. Int J Tuberc Lung Dis.

