# Supplementary figures and images for: Prevalence of Drug-Resistant Tuberculosis in Mainland China: Systematic Review and Meta-Analysis
Source: PLoS One. 2011 Jun 3;6(6):e20343. doi: 10.1371/journal.pone.0020343 (PMC3108589; doi:10.1371/journal.pone.0020343)

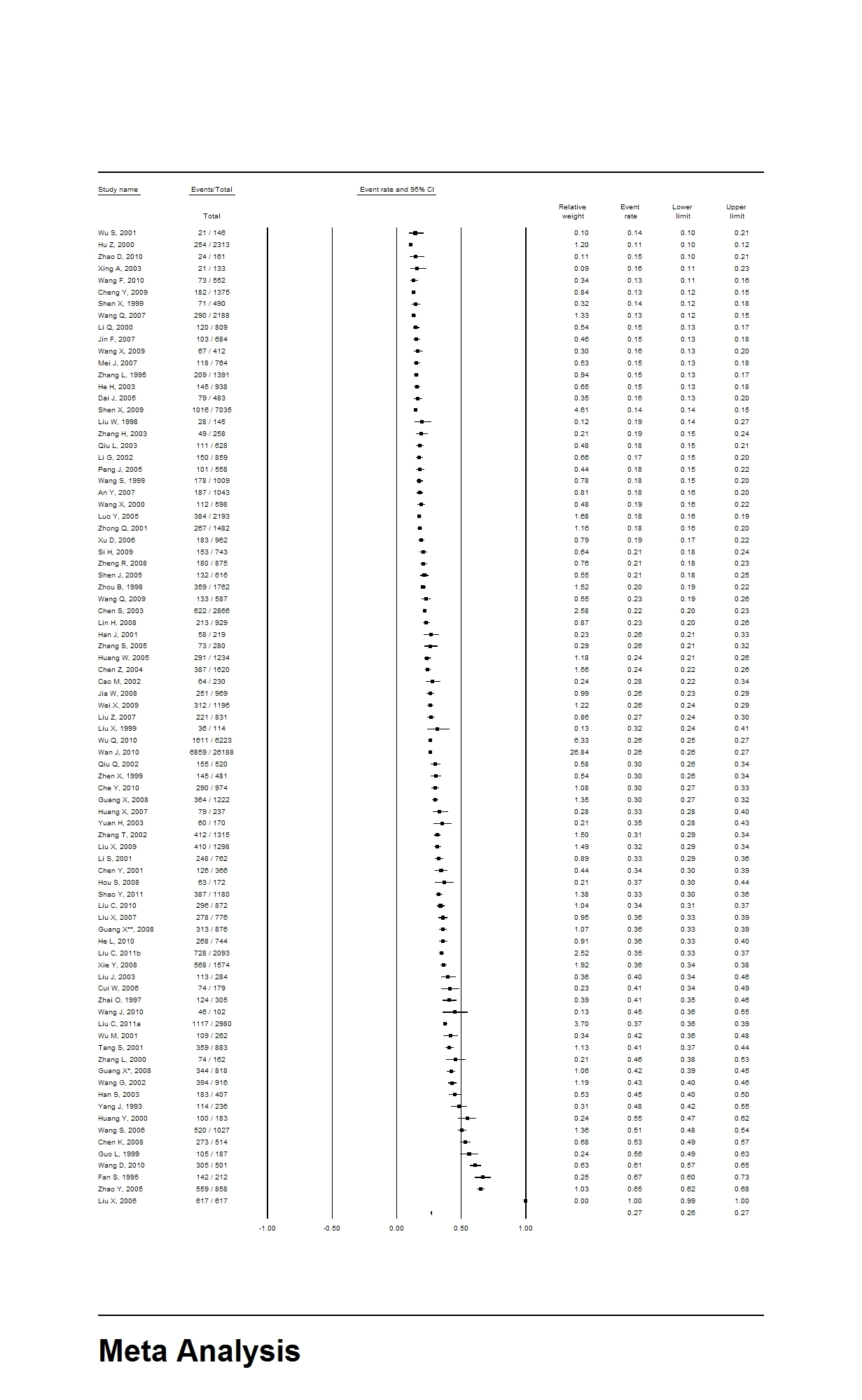

Supplement: Figure S1 — Forest plot of the meta-analysis on any drug resistance in new cases. (“*” in the figure means studies reported by the same publication). (TIF) [file pone.0020343.s001.tif]

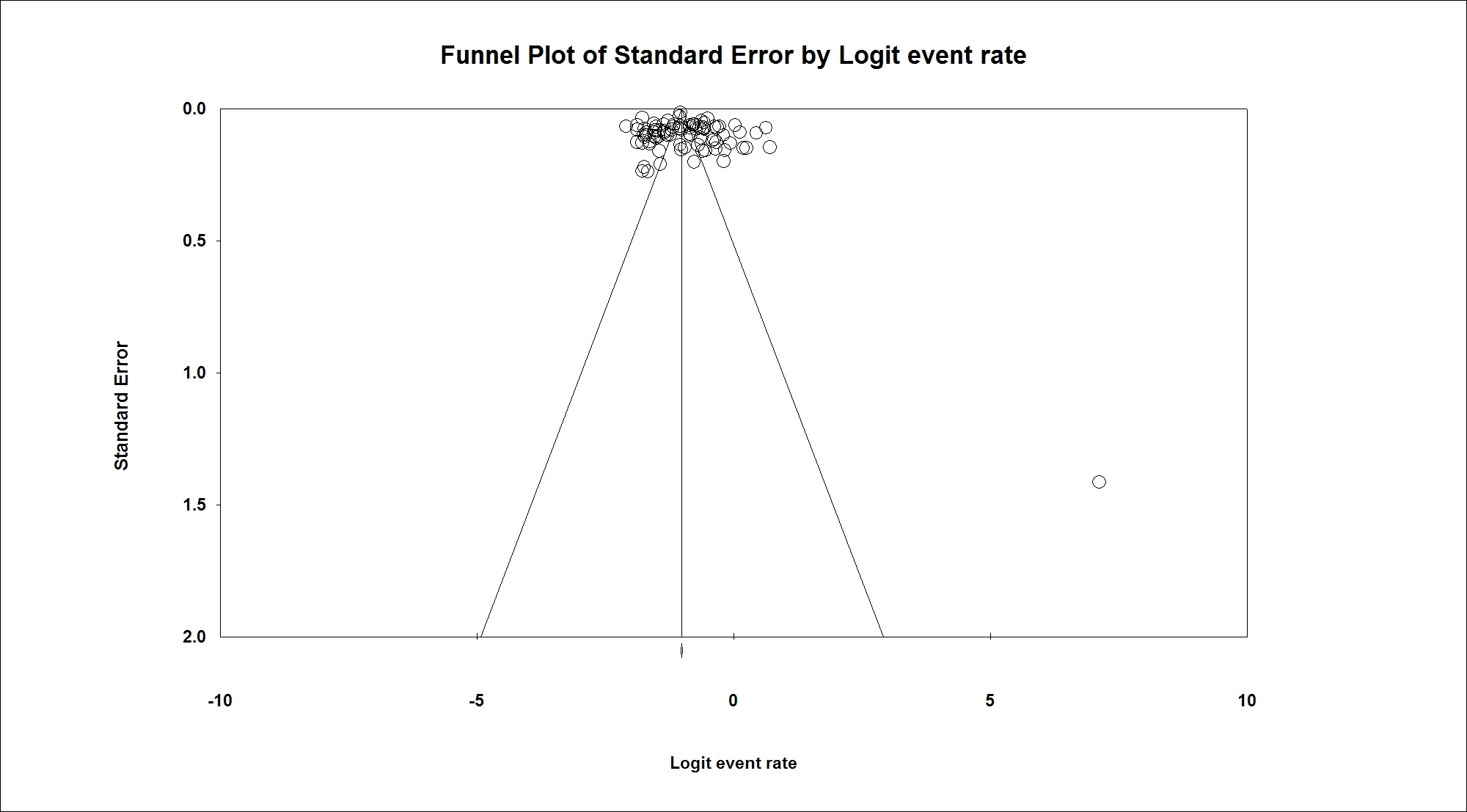

Supplement: Figure S2 — Funnel plot of the meta-analysis on any drug resistance in new cases. (TIF) [file pone.0020343.s002.tif]

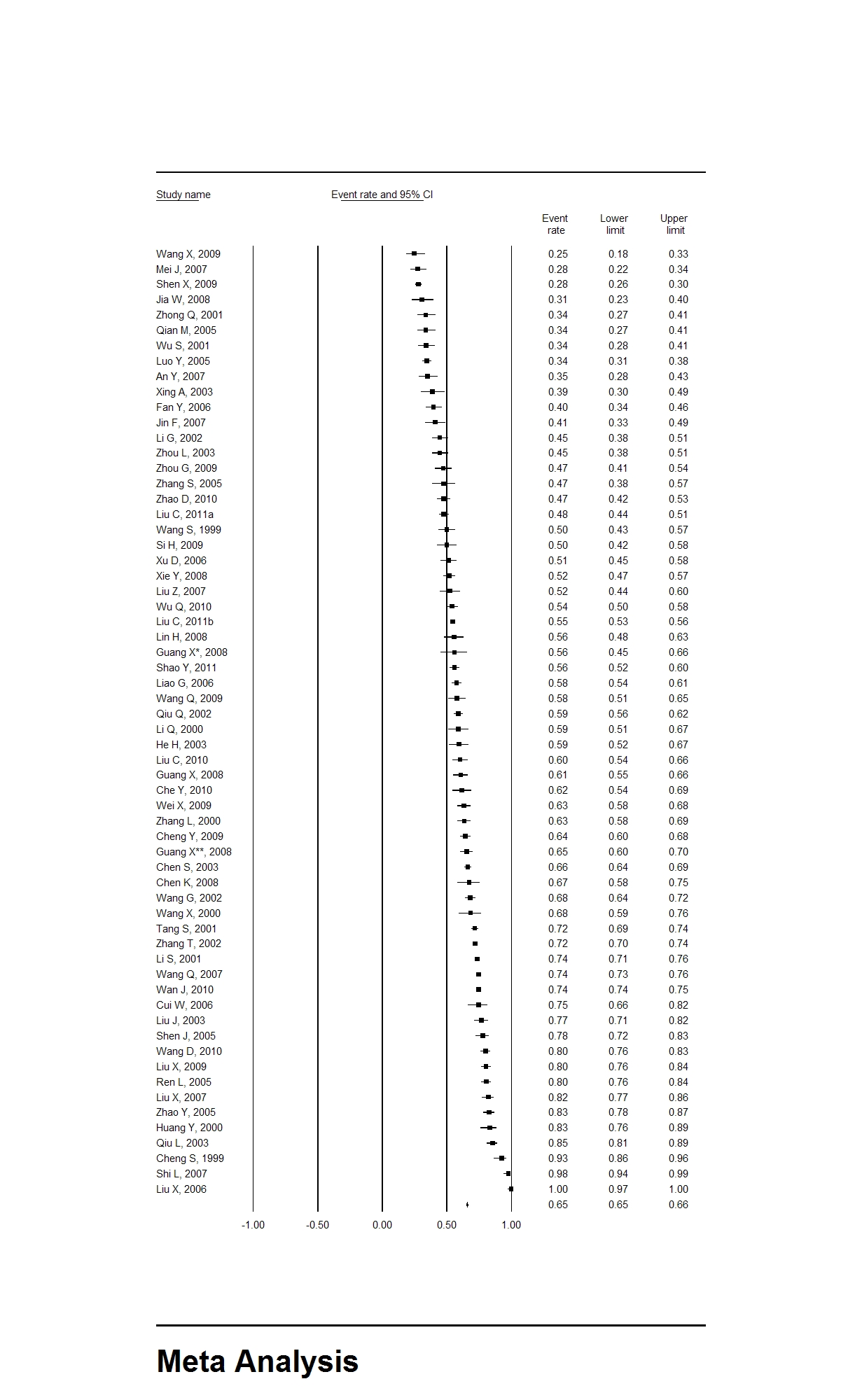

Supplement: Figure S3 — Forest plot of the meta-analysis on any drug resistance in previously treated cases. (“*” in the figure means studies reported by the same publication). (TIF) [file pone.0020343.s003.tif]

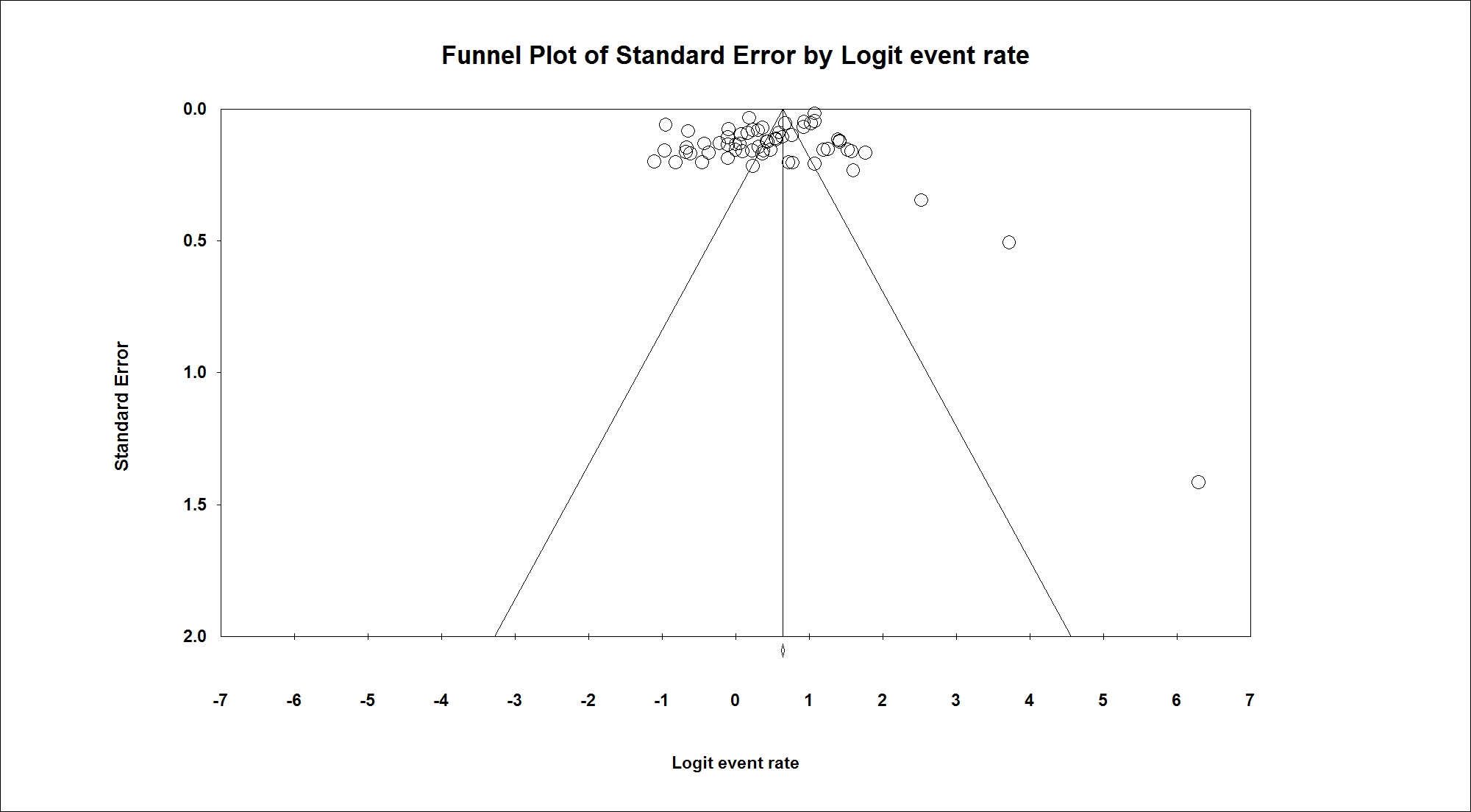

Supplement: Figure S4 — Funnel plot of the meta-analysis on any drug resistance in previously treated cases. (TIF) [file pone.0020343.s004.tif]
