# Supplementary material for: Prevalence of Drug-Resistant Tuberculosis in Mainland China: Systematic Review and Meta-Analysis
Source: PLoS One. 2011 Jun 3;6(6):e20343. doi: 10.1371/journal.pone.0020343 (PMC3108589; doi:10.1371/journal.pone.0020343)
Supplement: Table S1 — Included and excluded articles after full-text evaluation. (DOC) [file pone.0020343.s005.doc]

## Table S1. Included and excluded articles after full-text evaluation

**Part 1, included article:**

1. Yang J (1993) The trend of primary drug-resistant about tuberculosis in Chongqing. Sichuan Medical Journal 2: 76-77.

2. Fan S, Wei F, Guo L, Gao B, Jiang Z et al. (1995) Drug resistance determination of 212 new cases of pulmonary tuberculosis. The Journal of The Chinese Antituberculosis Association 3: 128-129.

3. Zhang L, Kan G, Tu D, Li J, Liu X (1995) Trend of initial drug resistance of tubercle bacilli isolated from new patients with pulmonary tuberculosis and its correlation with the tuberculosis programme in Beijing. Tubercle and Lung Disease 76: 100-103.

4. Zhai O, Wang X, Yang J, Yang X, Shao J et al. (1997) Primary drug-resistant analysis of 305 new tuberculosis cases. The Journal of The Chinese Antituberculosis Association 2: 95.

5. Liu W, Hao L, Zhang D (1998) The analysis of initial drug-resistant TB in 1993-1996. The Journal of The Chinese Antituberculosis Association 1: 23-24.

6. Zhou B, Guo W, Sun Y, Xiao X (1998) The tendency in development of initial drug-resistant TB in our hospital in 1978-1996. The Journal of The Chinese Antituberculosis Association 4: 176-178.

7. Cheng S (1999) The analysis of pulmonary tuberculosis drug resistance in the retreatment patients. Practical Preventive Medicine 3: 201-202.

8. Guo L, Ma J, Yuan J (1999) 187 cases of primary drug resistance of Mycobacterium tuberculosis. Shaanxi Medical Journal 11: 663-665.

9. Liu X, Mei L, Luo H (1999) 114 cases of initial drug resistance of Mycobacterium tuberculosis. The Journal of The Chinese Antituberculosis Association 2: 90.

10. Shen X, Xu R, Lv J (1999) Moniter of initial drug resistance in new cases of pulmonary tubercuslosis in Xicheng district, Beijing. The Journal of The Chinese Antituberculosis Association 1: 17-19.

11. Wang S, Zhang F, Duanmu H (1999) The prevalence of drug-resistant TB in Shandong Province. Chinese Journal of Tuberculosis and Respiratory Diseases 12: 728-730.

12. Zhen X, Yao L, Peng Y (1999) Surveillance of primary resistance in 481 cases of pulmonary tuberculosis. Chinese Journal of Tuberculosis and Respiratory Diseases 22: 362.

13. Hu Z, Cai H, Li L, Tu M (2000) Primary drug-resistance of Mycobacterium tuberculosis in Shanghai, 1985-1995. Chinese Journal of Tuberculosis and Respiratory Diseases 2: 82-84.

14. Huang Y (2000) Analysis on drug resistance in pulmonary tubercalosis in patients. Guangxi Medical Journal 22: 692-694.

15. Li Q, Wang X, He H (2000) WHO sample survey on drug resistant tuberculosis in Zhejiang, China. Chinese Journal of Tuberculosis and Respiratory Diseases 23　: 718.

16. Wang X, Q L, Han Y, Zhu Y, Yu D et al. (2000) The character of drug resistance of pulmonary tuberculosis cases in Zhejiang. The Journal of The Chinese Antituberculosis Association 22: 137-139.

17. Zhang L, Yang L, Zhao Y, Li A, Sun Z et al. (2000) High incidence of primary and required drug resistance of pulmonary tuberculosis in Henan Province. Chinese Journal of Health Laboratory Technology 10: 719-721.

18. Zhang Q, Xia X (2000) Analysis of drug resistance in new and retreatment TB patients. The Journal of The Chinese Antituberculosis Association: 31.

19. Chen Y, Liu L, Wu H, Li H, Zhang H et al. (2001) Primary drug resistance of Mycobacterium tuberculosis in Zhengzhou(1992-1996). The Journal of The Chinese Antituberculosis Association 3: 181-183.

20. Han J, Cao M (2001) Study on primary drug resistance of Mycobacterium tuberculosis in 3 years. Acta Universitatis Medicinalis Anhui 36: 154-155.

21. Li S, Zhao D, Sun Y, Jiang S (2001) The trend of drug resistance for in-patient pulmonary tuberculosis. The Journal of The Chinese Antituberculosis Association 23: 151-152.

22. Tang S, Xiao H, Xia X, Wu F, Sha W (2001) Study on drug resistance of Mycobacterium tuberculosis in different age group. Chinese Journal of Tuberculosis and Respiratory Diseases 24: 83-86.

23. Wu M, Wang X (2001) The analysis of primary drug resistant patients of TB. Journal of Clinical Pulmonary Medicine 6: 51.

24. Wu S, Xu C, Wang Y (2001) Analysis of drug resistance of 352 pulmonary tuberculosis cases. Journal of Clinical Pulmonary Medicine 6: 40.

25. Zhong Q, Qian M, Li J, Chen Q, Zhuo W et al. (2001) Study on drug resistance surveillance of tuberculosis in Guangdong province. The Journal of The Chinese Antituberculosis Association 23: 5-8.

26. Cao M, Han Q, Hu J, Xu Y, Zhang X (2002) Epidemic analyses of initial drug resistance in patients with pulmonary tuberculosis. Universitatis Medicinalis Anhui 37: 234-236.

27. Li G, Wu X, Zhang T, Zhou L, Li K et al. (2002) Surveillance of drug resistant tuberculosis in Hubei，China. Chinese Journal of Tuberculosis and Respiratory Diseases 25: 723-726.

28. Qiu Q, Song R (2002) Research on the trend of drug resistance of tuberculosis in Zaozhuang City in 1993-2001. Chinese General Practice 5: 641-642.

29. Zhang T, Luo K, Zhang X, Zhao G, Chen Y (2002) A study on the primary and acquired drug resistance of mycobacterium tuberculosis and the conditions of post-retreatment MDR. Journal of Guiyang Medical College 27: 487-491.

30. Wang G, Peng YL, Zhang G, Zhang L, Xing J et al. (2002) Sample survey of drug-resistant tuberculosis in Henan, China, 1996. Respirology 7: 67-72.

31. Chen S, Wang Z, Wu Y, Lin M (2003) Dynamic observation of drug resistance for tuberculosis. Modern Preventive Medicine 30: 182-183.

32. Han S, Wu M (2003) The analysis of 407 cases of primary drug resistance pulmonary tuberculosis. Journal of Clinical Pulmonary Medicine 8: 560-561.

33. He H, Yang S (2003) A Drug Resistance Analysis about 1103 Strains Tubercle Bacillus. The Journal of The Chinese Antituberculosis Association 25: 60.

34. Liu J, Zhang K, Wang Y, Zhang C, Xi C (2003) The analysis of 516 cases of drug resistance pulmonary tuberculosis in Qingdao City. The Journal of The Chinese Antituberculosis Association 25: 194-195.

35. Qiu L, Zhai O, Wang X, Wang X, Gao X et al. (2003) The situation of resistance to Anti-TB drugs among in patients in Shandong provincial thoracic hospital. The Journal of The Chinese Antituberculosis Association 25: 175-177.

36. Xing A (2003) A Drug Resistance Analysis about 236 cases clinical Tubercle Bacillus. Shanxi Medical Journal 32: 218-219.

37. Yang X, Lu X (2003) Research on the rend of drug resistance of the smear positive tuberculosis and tuberculosis control project. The Journal of The Chinese Antituberculosis Association 25: 219-222.

38. Yuan H (2003) The analysis of 170 cases of primary drug resistance pulmonary tuberculosisand and the effect of short-term chemotherapy in Peiyang City. The Journal of The Chinese Antituberculosis Association 25: 125.

39. Zhang H, Luo T, Zhu M, Ye F, He F et al. (2003) The analysis of 258 cases of primary drug resistance pulmonary tuberculosisand. The Journal of The Chinese Antituberculosis Association 25: 62.

40. Zhou L, Li G, Liu X, Li A, Liu X (2003) Anaylysis of drug resistance in 238 retreated smear-positive and sputum-culture-positive pulmonary tuberculosis cases. China Tropical Medicine 3: 756-757.

41. Chen Z, Tan S, Guan Y, Xiao F, Zhang Y et al. (2004) The drug resistant's dynamic monitoring of the first treated pulmonary tuberculosis patients in nine years. The Journal of The Chinese Antituberculosis Association 26: 121-122.

42. Wang Y, Chen J (2004) Surveillance of retreated drug resistant tuberculosis in Dalian City in 1993-2001. Chinese Journal of Public Health 20: 608.

43. Dai J, Kang X, Wu R, Wu Y, Kang X et al. (2005) of primary resistance of Mycobacterium tuberculosis in Shanghai Putuo district from 1996 to 2003. Laboratory Medicine 20: 467-469.

44. Huang W (2005) Development of TB drug resistance in Zhaoqing area of Guangdong Province. Journal of Clinical Pulmonary Medicine 10: 293-294.

45. Luo Y, Liu Z, Luo C, Liu Z, Luo C (2005) Analysis on situation of drug-resistant tuberculosis among outpatients in primary urban districts of Guangzhou from 1994-2003. The Journal of The Chinese Antituberculosis Association 27: 247-250.

46. Shen J, Yang W, Yang W (2005) The situation of resistance to anti-TB drugs among inpatients. The Journal of The Chinese Antituberculosis Association 27: 117-119.

47. Zhang S, Xu D, Yang J, Xu D, Yang J (2005) The analysis of drug resistance for pulmonary tuberculosis between migrant and resident. Modern Preventive Medicine 32: 1640-1641.

48. Zhao Y, Gao S, Yan G, Gao S, Yan G (2005) Surveillance of 10 drugs resistant tuberculosis in Henan Chest Hospital of China. Chinese Journal of Health Laboratory Technology 15: 405-406.

49. Cui W, Kong W, Gao P, Kong W, Gao P (2006) The analysis of drug resistance of 829 patients with pulmonary tuberculosis. Chinese Journal of Clinical Healthcare 9: 125-126.

50. Fan Y, Xiao H, Mei J, Xiao H, Mei J (2006) Analysis of drug resistance to antituberculosis drugs of the first time retreated pulmonary tuberculosis patients in Shanghai. Chinese Journal of Tuberculosis and Respiratory Diseases 29: 698-701.

51. Li G, Dong B, Wang F, Xu H (2006) Antimicrobial susceptibility test of the mycobacterium isolation strain of retreatment tuberculosis cases. Chinese Journal of Public Health 22: 1529.

52. Liao G, Lan H, Xu J, Lan R, Chen J et al. (2006) Study on drug resistance of mycobacterium tuberculosis of retreatment patients in different age. Guangxi Medical Journal 28: 1520-1522.

53. Liu X, Chen H, Lv C, Du Y, Wang S et al. (2006) Analysis of drug resistance of Mycobacterium tuberculosis in hospitalized tuberculosis cases from 1992 to 2004. The Journal of The Chinese Antituberculosis Association 28: 390-392.

54. Peng J, Yi L, Hu C, Liu Y, Li G (2006) Analysis on current situation of tuberculosis control project and initial drug resistance rate of pulmonary tuberculosis in Dongguan city. The Journal of The Chinese Antituberculosis Association 5: 330-332.

55. Qian M, Chen Q, Zhuo W, Jiang Y (2006) The analysis of 166 cases drug resistance pulmonary tuberculosis and the cause of that. Guangdong Medical Journal 11: 1552-1553.

56. Ren L, Gui X, Xia X, Tang S, Wu X et al. (2006) The investigation on drug resistance of hospitalized previously retreated tuberculosis patients during 1996-2001 in Shanghai Pneumology Hospital. The Journal of The Chinese Antituberculosis Association 5: 310-312.

57. Wang S, Li Y, Niu G (2006) Survey of initial drug resistant in patients with pulmonary tuberculosis. The Journal of The Chinese Antituberculosis Association 1: 43-44.

58. Xu D, Zhang S, Yang J, Zhang S, Yang J (2006) The analysis on the trend of drug resistance of tuberculosis in Huzhou city, Zhejiang. The Journal of The Chinese Antituberculosis Association 28: 103-105.

59. Huang X, Xiao X, Ye Y (2007) Study on primary drug resistance of retreated mycobacterium tuberculosis in Maoming City of Guangdong province. The Journal of The Chinese Antituberculosis Association 29: 174-175.

60. Jin F (2007) Analysis of 833 cases drug resistance of Mycobacterium tuberculosis in Shaoxing towndown. Modern Journal of Integrated Traditional Chinese and Western Medicine 16: 1385-1386.

61. Liu X, Gao G, Chen H, Li Z, Lv C et al. (2007) Analysis on drug resistance of Mycobacterium tuberculosis in hospitalized patients from 2004 to 2006. Hebei Medical Journal 29: 1260-1261.

62. Liu Z, He H, Miu Z, Li Q (2007) Analyzing 984 drug resistant Mycobacterum Tuberculosis. The Journal of The Chinese Antituberculosis Association 29: 167-170.

63. Mei J, Shen X, Shen M, Gui X, Sun B (2007) Survey of drug resistant tuberculosis in Shanghai, China. The Journal of The Chinese Antituberculosis Association 29: 395-398.

64. Shi L, Lang Z, Wei S, Wan K, Lang Z et al. (2007) Analysis on drug resistance of 169 cases of pulmonary tuberculosis failing to respond to retreatment in Tibet of China. surveillance of diseases 22: 388-389.

65. Wang Q (2007) Analysis on drug resistance of tuberculosis in hospitalized patients. Chinese Journal of Public Health 23: 692-693.

66. Chen K (2008) Drug resistance of tuberculosis in Haikou City. China Tropical Medicine 8: 1805-1806.

67. Hou S, Fang H, Song X, Ding S (2008) Culture and analysis on primary drug resistance of mycobacterium tuberculosis. Zhejiang Clinical Medical Journal 10: 552.

68. Lin H, Liu J, Chen L, Wang Z, Jing K et al. (2008) Drug resistance of tuberculosis from 2003 to 2006 in Chongqing. Acta Academiae Medicinae Militaris Tertiae 30: 1183-1185.

69. Zheng R, Zhang X, Huang C, Zhang X, Huang C (2008) Analysis of the drug resistance of 875 mycobacterium tuberculosis strains. Modern Preventive Medicine 35: 4697-4698.

70. He G, Zhao Y, Jiang G, Liu Y, Xia H et al. (2008) Prevalence of tuberculosis drug resistance in 10 provinces of China. BMC infectious diseases 8: 166.

71. Jia W, Wu W, Zhang W, Gu X, Yu J et al. (2008) The report of WHO drug resistance surveillance on tuberculosis, Xinjiang. The Journal of The Chinese Antituberculosis Association: 307-310.

72. He G, Zhao Y, Jiang G, Liu Y, Xia H et al. (2008) Prevalence of tuberculosis drug resistance in 10 provinces of China. BMC infectious diseases 8: 166.

73. Liu X, Chen H, Lv C, Li G, Cui Q et al. (2009) Study on drug resistance of Mycobacterium tuberculosis in hospitalized patients in different age group from 2005 to 2008. Hebei Medical Journal 31: 3302-3304.

74. Wang Q (2009) The analysis of drug resistance of pulmonary tuberculosis in Shengli oil field. Journal of Clinical Pulmonary Medicine 14: 480-482.

75. Wang X, Wu M, Shen X (2009) Analysis on drug resistance of Mycobacterium tuberculosis in hospitalized patients in Suzhou City. Journal of Clinical Pulmonary Medicine 14: 1674.

76. Cheng Y, Zeng X, Wu Y (2009) Dynamic analysis of the status of drug resistance of Mycobacterium tuberculosis of pulmonary tuberculosis cases from 2001 to 2005 in Quanzhou. The Journal of The Chinese Antituberculosis Association: 68-70.

77. Si H, Li Q, Mu T, Liang C, Yang Q et al. (2009) Analysis of anti-TB drug resistance surveillance in Linxia, Ganan and Dingxi in Gansu province. The Journal of The Chinese Antituberculosis Association: 206-209.

78. Zhou G, Cui J, Yu N (2009) Analysis of secondary drug-resistant tuberculosis 104 cases. Chinese Journal of Misdiagnostics: 3676-3677.

79. Wang D, Yang C, Kuang T, Lei H, Meng X et al. (2010) Prevalence of Multidrug and extensively drug-resistant tuberculosis in Beijing, china: A hospital-based retrospective study. Japanese Journal of Infectious Diseases 63: 368-371.

80. Wang J (2010) Study on drug resistance of Mycobacterium tuberculosis in hospitalized patients. Journal of Clinical Pulmonary Medicine 15: 703.

81. Wei X, Wu S (2009) Analysis on the Drug Resistance of 1 537 Cases of Mycobacterium Tuberculosis. Chinese Journal of Medicinal Guide 9: 1553-1555.

82. Che Y, Yu M, Ping G (2010) Analyzing 1149 drug resistant M. tuberculosis. Chinese Journal of Health Laboratory Technology: 1494-1495.

83. He L, Zhu J, Zhao Z (2010) Analysis on Drug-resistant Strains of 268 TB Patients with Initial Treatment in Shijiazhuang City. Occup and Health 26: 67-69.

84. Liu C, Zhou Y, Tan N, Guan F, Lin Y et al. (2010) Analysis on Drug-resistant TB Strains in Jiangyang City of Guangdong Province. The Journal of The Chinese Antituberculosis Association: 606-608.

85. Liu L, Luo K, Zhang T, Wang Y, Chen H et al. (2010) Analysis on clinical drug resistance of multi-drug resistant Mycobacterium tuberculosis. Laboratory Medicine 25: 683-685.

86. Wang J, Liu Y, Wu D (2010) Investigation analysis on drug tolerance of initial treatment pulmonary TB sufferers hospitalized from 2007 to 2008. Chongqing Medical Journal 39: 2944-2945.

87. Wang F, Zhu Q, Fei F (2010) Analysis of primary drug resistance of 552 strains of Mycobacterium tuberculosis in Qingpu district. Journal of Clinical Pulmonary Medicine 15: 810-811.

88. Wu Q, Tan W, Yang Y, Luo D, Xu L et al. (2010) Report on the surveillance of drug-resistant resistant tuberculosis in Shenzhen，1999-2008. Journal of Clinical Pulmonary Medicine 15: 1121-1123.

89. Zhang Z, Xiao H (2010) Clinical study on pulmonary tuberculosis with first retreatment in Shanghai Pulmonary Hospital. The Journal of The Chinese Antituberculosis Association: 1-5.

90. Zhao D, Zhao Y, Li H, Yan G, Yang H et al. (2010) Analysis on characteristics of drug resistance of Mycobacterium tuberculosis isolated in 2007 - 2009 in some areas of Henan. Chinese Journal of Health Laboratory Technology: 1109-1111.

91. Liu C, Li H, Li L, Hu L, Wang Q et al. (2011) Anti-tuberculosis drug resistance patterns and trends in a tuberculosis referral hospital, 1997-2009. Epidemiol Infect: 1-10.

92. Shao Y, Yang D, Xu W, Lu W, Song H et al. (2011) Epidemiology of anti-tuberculosis drug resistance in a chinese population: current situation and challenges ahead. BMC Public Health 11: 110.

93. Liu C, Liu Y (2011) Analysis of drug-resistance on 3653 sample of tuberculosis patients. Modern Preventive Medicine: 725-727.

94. Liu C, Li H, Li L, Hu L, Wang Q et al. (2011) Anti-tuberculosis drug resistance patterns and trends in a tuberculosis referral hospital, 1997-2009. Epidemiol Infect: 1-10.

95. Shao Y, Yang D, Xu W, Lu W, Song H et al. (2011) Epidemiology of anti-tuberculosis drug resistance in a chinese population: current situation and challenges ahead. BMC Public Health 11: 110.

**Part 2, excluded articles:**

**Incorrect data:**

1. Zhao Y, Zhang L, Li Y (2001) Analysis of aged Tuberculosis patients drug resistance of 10 anti-TB drugs. Buccetin of Chinese Antituberculosis Assoclation 23: 261-262.

2. Lin Y, Xu D, Wang Z (2008) Analysis On Drug Resistance of Inpatients With Tuberculosis In Nanning. Modern Preventive Medicine 35: 4686-4687, 4690.

**Based on the same population:**

1. Liu X, Chen H, Wei X, Lv C, Du Y (2010) Analysis of drug resistent result. Hebei Medical Journal 32: 2112-2113.

2. Che Y, Yu M, Ping G (2010) Analysis of drug resistance of 974 new tuberculosis cases in Ningbo, Zhejiang. Disease Surveillance: 614-616.

3. Xu L, Yang Y, Wu Q, Guan H, Li M (2010) Analysis of first l ine anti-TB drug resistance surveillance in Shenzhen. The Journal of The Chinese Antituberculosis Association: 204-207.

4. Xu L, Yang Y, Wu Q, Li M, Zhang Y, et al. (2010) Analysis on situation and pattern of drug resistance tuber culosis in suspected multi-drug resistance patients in Shenzhen. The Journal of The Chinese Antituberculosis Association: 318-322.

5. Xu L, Yang Y, Lv D, Wu Q, Li M, et al. (2010) Analysis of the situation of anti-TB drug resistance and its risk factors in Shenzhen City. China Tropical Medicine 10: 1333-1335.

6. Liang X, Yu X, Zheng X (2010) Analysis of 364 strains mycobacterium tuberculosis resistance. Chinese Journal of Misdiagnostics 10: 2408-2409.

7. Yang L, Yuan X (2009) Analysis of resistance pattern of 88 strains of isoniazid-resistant Mycobacteria. International Journal of Laboratory Medicine 30: 1058-1059, 1062.

8. Che Y, Yang W, Yu Y (2009) Sample survey of drug-resistant tuberculosis in Ningbo, 2007. The Journal of The Chinese Antituberculosis Association: 53-54.

9. Deng Q, Zhan N, Cai X, Ceng J, Fang M, et al. (2007) Analysis of drug resistence of 229 mycobacterium tuberculosis strains in shengzhen east lake Hospita. Journal of Clinical Pulmonary Medicine 12: 5-6.

10. Miu Z, Li Q, He H, Liu Z, Yang S, et al. (2007) Developing status and tendency of drug resistant tuberculosis in Zhejian. The Journal of The Chinese Antituberculosis Association 29: 215-218.

11. Lv K, Liao G (2006) Treatment effect of antituberculosis drug resistance and multi-drug resistance of TB inpatients. Journal of Clinical Pulmonary Medicine 11: 307-308.

12. Huang X, Luo D, Ouyang Z, Long Q (2005) Evaluation of Drug-resistance to antituberculosis Drugs in Shenzhen. China Pharmacy 16: 1084-1085.

13. Wang Z, Chen S, Cheng Y (2005) Dynamic observation of drug resistance for tuberculosis in Quanzhou. Journal of Clinical Pulmonary Medicine 10: 755-756.

14. He Z, Yi S, Chen H, Wu P (2003) Multi-drug resistance of TB in Hunan Province in 1996-2001. Practical Preventive Medicine 10: 53-55.

15. Song R, Qiu Q, Kong W (2003) The analysis of drug resistance pulmonary tuberculosis in Zaozhuang City. The Journal of The Chinese Antituberculosis Association 25: 49-50.

16. Zhong J, Mai J, Tang H, He L, Huang P (2002) Monitoring of initial drug resistant status in patients with pulmonary tuberculosis in Haizhu District of Guangzhou City. China Tropical Medicine 2: 390-392.

17. Lin H (2001) The investigation of primary drug resistance of TB in Futian District, Shenzhen. Journal of Guangdong College of Pharmacy 17: 246.

**Duplicate publications:**

1. Ma J, Guo L, Yuan J (1999) Primary drug resistance of Mycobacterium tuberculosis of 187 cases. Journal of Xinxiang Medical College 2: 156-157.

2. Wang S, Li Y, Niu G (2006) Monitoring of initial drug resistance tuberculosis patients hospitalized. Journal of Clinical Pulmonary Medicine 11: 239-241.

3. Zhang L, Tu D, Li J, Liu X (1994) Trend of primary drug resistance tuberculosis in Beijing. The Journal of the Chinese Antituberculosis Association 1: 14-16.

**Small sample size:**

1. Wu G, Jiang J (2010) Analysis characteristic of 209 cases of drug resistance mycobacterium tuberculosis. Hainan Medical Journal: 109-110.

2. Chen X, Zheng M, Zhong L (2010) Analysis on Drug Resistance of 300 Cases Pulmonary Tuberculosis. Hebei Medicine 16: 172-174.

3. Wang D, Zhang H, Wang J, Sun X (2010) Analysis on drug-resistance of tuberculosis in sputum of 123 TB pafien in Binzhou city of Shandong province. Journal of Clinical Pulmonary Medicine 15: 1256.

4. Cao H, Wu C, Mi L, Liu P, Chen W, et al. (2010) Preliminary Investigation on the MDR-TB and XDR-TB of MTB Clinical Isolates From Xinjiang Area. Progress in Modern Biomedicine: 890-892.

5. Sun D, Chen Y (2010) Analysis of 156 patients hospitalized with drug resistance tuberculosis bacili. The Journal of The Chinese Antituberculosis Association: 470-471.

6. Che Y, Yu M, Ping G (2010) Drug resistance in patients of pulmonary tuberculosis in different periods. Chinese Journal of Preventive Medicine: 947-949.

7. Wu Q, Liu R, Wu Q (2010) Analysis of drug-resistant tuberculosis in 296 elderly inpatients in Tianjin. Chinese Journal of Geriatrics: 811-813.

8. Zhang H, Zhang A, Zhao P, Zhang S, Zhang S, et al. (2009) Analysis of the status and risk factors of drug resistant tuberculosis in Chaoyang district in Beijing. The Journal of The Chinese Antituberculosis Association: 218-222.

9. Che Y, Yu M, Ping G, Yu Y (2009) Analysis of 418 strains mycobacterium tuberculosis resistance. Chinese Journal of Health Laboratory Technology: 1178-1179.

10. Guo L, Ma J, Wang M, Wang J (1996) Determination about primary drug resistance in mycobacterium tuberculosis from first visit pulmonary tuberculosis type Ⅲ. Journal of xinxiang medical college 4: 374-376.

11. Wang Z, Chen S, Wu H (1997) Analysis of 176 Mycobacterium tuberculosis drug resistance on the determination of ofloxacin. The Journal of the Chinese Antituberculosis Association 2: 96-97.

12. Ding H (1998) Analysis of 2578 strains resistance of 5 kinds of anti-TB drugs. Journal of Clinical Laboratory Science 2: 111-112.

13. Yue L, Wang Y, Zhang K (1998) Drug resistance in 134 aged patients with pulmonary tuberculosis. Chinese Journal of Geriatrics "": 332.

14. Zhang J (1999) The incidence of multiple drug-resistant TB. Practical Preventire Medicine 3: 193.

15. Zhang X, Qu H, Liu S, Li R (1999) Analysis tuberculosis initial drug resistance and secondary drug resistance in Zouping. China Public Health 11: 1031.

16. Zhang Y (1999) Analysis of 126 cases of drug resistance of tuberculosis patients. Journal of Clinical Internal Medicine 16: 275.

17. Liao Y, Wei Q, Xian H, Gong L (2000) Study of complex, intractable pulmonary tuberculosis patients and L-TB drug resistance in Mianzhu City. Modern Preventive Medicine 27: 112-113.

18. Hu Q (2001) Causes of 105 cases of drug-resistant TB. Chinese Journal of Medicine 36: 44-45.

19. Cheng Z, Liu D (2001) Analysis of primary and re-treatment 203 patients with pulmonary tuberculosis resistant. Shanxi Medical Journal 30: 415-416.

20. Wang T, Zhang Y, Zhang H (2001) Survey and Analysis on Drug Resistance of 189 Hospitalized Patients with Pulmonary Tuberculosis. Herald of Medicine 20: 449-450.

21. Deng D, Liu Y, Duan W, Yang J (2002) Analysis of 76 cases of drug resistance tuberculosis. Sichuan Medical Journal 23: 503-504.

22. Liang S, Li Y, Cao F (2002) 51 cases of drug resistance in tuberculosis patients and the treatment of MDR-TB. Guangxi Medical Journal 24: 423-424.

23. Liu M (2002) 83 cases of drug resistance in primary pulmonary tuberculosis. Guangxi Medical Journal 24: 822-823.

24. Huang L, Wu W (2002) Analysis of 135 cases of drug resistance in tuberculosis. Sichuan Medical Journal 23: 404-405.

25. Kong X, Yang S, Zhang G, Sun F, Li B, et al. (2002) TB drug resistance survey in Yichang City. Chinese Journal of Disease Control and Prevention 6: 166-167.

26. Xu G, An D, Zhao M, Yang W, Wang J (2002) A Surveillance Report on the Drug - resistant Tuberculosis in Ningbo of Zhejiang Province. China Preventive Medicine 3: 108-110.

27. Xu Q, Lu X, Kong Q, Liu X (2002) Analysis of drug resistance of mycobacterium tuberculosis patients in Shengli Oilfield. Buccetin of Chinese Antituberculosis Assoclation 24: 361.

28. Li W, Zhang C, Zhu L, Kuang T (2003) Mycobacterium tuberculosis strain type identification and analysis of drug resistance. People s Military Surgeon 46: 574-576.

29. Zhang H (2003) Analysis of TB drug resistance tuberculosis patients in rural areas of Shijiazhuang. Modern Jorunal of Integrated Traditional Chinese and Western Medicine 12: 2439-2440.

30. Liang Q, Chen Q, Lin Y, Qiu Z, Wang W (2003) Analysis of 200 cases of tuberculosis drug resistance. Journal of Preventive Medicine Information 19: 50-51.

31. Xu D, Li J, Gu J (2003) Analysis of primary and re-treatment drug resistance of tuberculosis in Huzhou City. Buccetin of Chinese Antituberculosis Assoclation 25: 45.

32. Ai Y, Huang Q, Li G, Wu M, Cheng Y, et al. (2004) Surveillance of drug resistant tuberculosis in Huangshi of China. Bulletin of Chinese Antituberculosis Association 26: 230-232.

33. Wang G, Li X, Chen Q, Zheng X, Lin Z, et al. (2004) Analysis on the drug resistance of 1293 tuberculosis mycobacterium strains. Occupation and Health 6: 41-43.

34. Wang Z (2004) Analysis of resistance of 71 tubercle bacillus strains to antibiotics. China Tropical Medicine 4: 75-76.

35. Wang J, Zhao J, Chen Z, Li Z (2004) Analysis on drug resistance of 361 cases with pulmonary tuberculosis in nanshan region. Modern Preventive Medicine 31: 167-168, 171.

36. Duan H, Yang S, Guo D (2005) Analysis of 160 cases of TB drug resistance in sputum of pulmonary tuberculosis. Journal of Shanxi Medical University 36: 88-89.

37. Han X, Huang Z (2005) Analysis of drug resistance spectral of 300 cases of tuberculosis. Journal of Clinical Pulmonary Medicine 10: 460.

38. Miao Y, Duan H, Du C, Zhang X, Xiu L (2005) Analysis of 57 cases of pulmonary tuberculosis drug resistance. Journal of Shanxi Medical University 36: 467-468.

39. Pan J, Yang R, Zhao W (2006) The Analysis of Drug Resistance in Military Patients with Pulmonary Tuberculosis. Clinical Journal of Medical officer 34: 462-464.

40. Qian M, Wu H (2006) The result of re-treatment drug resistance of 88 cases with sputum positive pulmonary tuberculosis patients. South China Journal of Preventive Medicine 32: 37-38.

41. Yao Y, Yang X (2006) Resistance and efficacy of treatment to the second Anti-TB drugs retreated patients between the city area and secluded mountain area. Journal of Clinical Pulmonary Medicine 11: 313-314.

42. Li L, Huan J, Xu L, Chen L (2006) Analysis of the local strains of Mycobacterium tuberculosis drug resistance in Yunnan Province. Practical Preventive Medicine 13: 642-643.

43. Wen H, Fan M, Zhang J, Yan J, Li C (2006) Actualities of tubercule bacillus drug-resistance in Taiyuan Tuberculosis Hospital. Shanxi Medical Journal 35: 675-676.

44. Yang B, Xu B, Jiang W, Zhou P, Jiang Q (2006) Analysis of Drug-Resistant Mycobacterium tuberculosis Isolated from the Rural Area in North Jiangsu Province. Journal of Tropical Medicine 6: 409-411, 378.

45. Yang H, Cao J, Wang H, Kang S, Zhang Y (2007) Identification and testing of 182 cases of type strains of mycobacterium drug resistance. Journal of Clinical Pulmonary Medicine 12: 1407.

46. Pei D, Wang S, Wang X, Wang J, Wang Y (2007) Analysis On Drug Resistance of Mycobacterium Tuberculosis. Modern Preventive Medicine 34: 1429-1431.

47. Shen X, Shen M, Gui X, Gao Q, Mei J (2007) The prevalence and risk factors of drug-resistant tuberculosis among migratory population in Shanghai,China. Chinese Journal of Tuberculosis and Respiratory Diseases 30: 407-410.

48. Xing G (2008) Analysis of patients with drug resistant tuberculosis. Journal of Medical Forum 29: 68-69.

49. Ding H, Chen Y (2009) Surveillance of 10-drug-resistant tuberculosis in Nanjing Chest Hospital. Acta Universitatis Medicinalis Nanjing(Natural Science) 10: 1361-1364.

50. Zhang Y, Wang Y, Luo K, Xiong M, Long Y, et al. (2009) An Analysis on Resistance Status of 2327 Strains of Bacillus tuberculosis to Multi-drugs. Journal of Guiyang Medical College 34: 643-645.

51. Wang Y, Liu Y, Liu X, Tian H (2009) Drug resistance of Mycobacterium tuberculosis in Yan an. Journal of Shanxi Medical University 40: 58-60.

52. Xu Y, Wang L, Zhu Y, Su X (2009) Analysis of drug resistance of Mycobacterium tuberculosis isolated from sputum of 202 patients with pulmonary tuberculosis. Chinese Journal of Infection Control 8: 14-17.

53. Li J, Lv Y, Chen W (2010) Analysis of tuberculosis drug resistance patients from 1999 to 2007 in Luohu District, Shenzhen. Journal of Clinical Pulmonary Medicine 15: 411-412.

54. Zhang X, Zhao P, Zhang S, Zhang X (2010) The investigation and analysis of the current status of drug-resistant tuberculosis among migratory population in Chaoyang district in Beijing. Chinese Journal of Laboratory Medicine 33: 30-32.

**Required data unavailable:**

1. Zhou X, Yu Y, Dang Y (1995) Analysis of 82 cases sputum Mycobacterium tuberculosis drug resistance. The Journal of the Chinese Antituberculosis Association 3: 111.

2. Kuang T, Jin G, Song P, Li W, Wang Z (1998) An investigation on multiple drug resistance of the rifampin resistant strains of mycobacterium tuberculosis. Acta Microbiologica Sinica 2: 152-154.

3. Zhang L, Zhai B, Zhang Y, Ren R, Zhao Z (1998) 1483 cases of TB drug resistance test results. Hebei Medical Journal 4: 242-244.

4. Zhao Q (1998) Analysis of multiple drug-resistant tuberculosis 133 cases. Zhejiang Medical Journal 6: 360-362.

5. Hu Z, Cai H, Li L, Tu M (1999) Analysis of the identification of Mycobacterium species in strians during the period 1987～1996 in Shanghai. Buccetin of Chinese Antituberculosis Assoclation 1: 34-36.

6. Wang G, Peng Y, Zhang G, Xing J, Li D, et al. (1999) WHO report on TB drug resistance surveillance of Henan Province. Buccetin of Chinese Antituberculosis Assoclation 3: 167-168.

7. Zhang J, Zhang J, Gao W, Na X, Hu F, et al. (1999) Analysis of ofloxacin(OFLX)resistance of in patients with pulmonary tuberculosis. Buccetin of Chinese Antituberculosis Assoclation 1: 24-25.

8. Zhang G, Liu S (2000) Determination of drug resistance of retreatment bacteria-positive pulmonary tuberculosis in 1994-1997 in Haiyang City, Shandong Province. Occupation And Health 9: 19-20.

9. Liu Z (2001) Analysis drug resistance of TB from 1993 to 1997 in four old city of Guangzhou City. Buccetin of Chinese Antituberculosis Assoclation 23: 109.

10. Zhang L, Zheng W, Huang X (2001) Haiyan tuberculosis drug resistance surveillance report. Practical Preventire Medicine 8: 202-203.

11. Hu H, Peng Y, Zhang C (2001) Survey of TB drug resistance in Yuzhou. China Public Health 17: 622.

12. Wang M, Zhao J, Chen Y (2001) Analysis of 50 cases of tuberculosis retreatment cases and primary drug resistance. Occupation and Health 17: 142-143.

13. Huang J, Wu B, Zhang Y (2002) Clinical analysis of the results of 182 cases of resistant Mycobacterium tuberculosis. Jiangsu Medical Journal 6: 457-458.

14. Liu R, Guo H (2002) Analysis of 375 resistances of mycobacterium tuberculosis and causes. China Public Health 18: 443.

15. Liu Y, Duan W, Xiong G, Wu J, Cao H, et al. (2002) The Dynamic Chang and Drug Resistance of Tuberculosis in Sichuan Province. Journal of Preventive Medicine Information 18: 9-10.

16. Zhang Y, Fu W, Zhu G, Chen D, Wang L, et al. (2002) Tuberculosis Drug Resistance in Shaoxing. Chinese Journal of Preventive Medicine 36: 120.

17. Li G, Zhou L, Liu X, Li A, Li K, et al. (2003) Study on Drug Resitance of Mycobacterium Tuberculosis in Different Age Group and Time of Anti-tuberculosis Medications in Hubei Province. The Journal of The Chinese Antituberculosis Association 25: 36.

18. Zhu J (2003) Analysis resistance characteristics of tuberculosis patients in hospital in Jinhua. Bulletin of Chinese Antituberculosis Association 25: 89.

19. Jiang Y, Yu Z, Yin H, Han Y (2004) Analysis of 259 cases of pulmonary tuberculosis drug resistance. Bulletin of Chinese Antituberculosis Association 26: 190-191.

20. Wang X, Zhao Y, Wang X (2004) Results of epidemiological survey of tuberculosis in rural areas of Pingdingshan. China Tropical Medicine 4: 370-371.

21. Yang B, Xu B, Jiang W, Zhou P, Jiang Q (2004) Study on the epidemiology and determinants of drug--resistant tuberculosis in northern rural area of Jiangsu province. Chinese Journal of Epidemiology 25: 582-585.

22. Zuo Y, Wang H, Li Y (2004) Analysis of 2537 cases of pulmonary tuberculosis drug resistance. Shandong Medical Journal 44: 27-28.

23. Huang J (2004) 54 cases of pulmonary tuberculosis patients with drug resistance determination. Guangxi Medical Journal 26: 95.

24. Wang X, Zhao Y, Song S, Chen X, Yu C, et al. (2005) TB drug resistance study of second-time treatment of TB-positive patients. Journal of Clinical Pulmonary Medicine 10: 698-699.

25. Zhao J, Wang Q, Wang J, Xu P (2005) Surveillance On Drug Resistance of Mycobacterium Tuberculosis In Nansha District. Modern Preventive Medicine 32: 132-134.

26. Xiao Y, Liu Y, Ai L, Wang H, Zhao X, et al. (2005) Investigation and analysis of 204 cases TB drug resistance. Guangdong Medical Journal 26: 394-395.

27. Huang B, Qiu Y, Xiao J, Peng S, Wen G (2006) Study On Drug Resistance of Mycobacterium Tuberculosis In Baoan District. Modern Preventive Medicine 33: 538, 542.

28. Wang W, Li H, Wang A, Wu X, Li S (2006) Clinical Analysis of Makeup of Pathogene and Resistance Drug on 69 Patients with Retreatment Pulmonary Tuberculosis. West China Medical Journal 21: 248-249.

29. Hua J, Xu L, Li L (2006) Analysis of Mycobacterium tuberculosis drug resistance in Yunnan Province. Practical Preventive Medicine 13: 911-912.

30. Li J, Chen X (2006) Analysis of retreatment cases of TB drug resistance in Dalian City. Occupation and Health 22: 2220-2222.

31. Xu L, Wu Y, Ming L, Yan X (2007) Survey on multiple drug-resistance to mycrobacteria in Changzhi region. Journal of Shanxi Medical University 38: 715-718.

32. Zhang Z, Cui Y, Gao T, Zhao F, Du C (2007) Analysis of initial drug resistance of tuberculosis patients from 2004 to 2005 in Changping District, Beijing. Bulletin of Chinese Antituberculosis Association 29: 96-97.

33. Tan Y, Yao M, Yi S, Tan X (2008) Drug Resistance of Mycobacterium Tuberculosis Clinical Isolates in Hunan Province,2001-2006. China Preventive Medicine 9: 193-196.

34. Yang X, Wang Z, Du Z (2008) Analysis of TB drug resistance of Mycobacterium tuberculosis. Chinese Journal of Health Laboratory Technology 18: 2426.

35. Zhang X, Liu Z, Zeng L, Zhao X, Wang X, et al. (2008) Pilot study on drug-resistance of Mycobacterium tuberculosis in Xi an city,Shaanxi province,China. Disease Surveillance 23: 637-639.

36. Xing J (2008) 40 cases of XDR-TB sputum TB culture and sensitivity test of a preliminary assessment report. Chinese Journal of Misdiagnostics 8: 8337.

37. Tan Y, Yi S, Liang Z (2009) Investigation on the drug resistance status of 591 strains Mycobacterium tuberculosis. The Journal of The Chinese Antituberculosis Association: 157-159.

38. Xing G, Ma Z (2009) Analysis of 166 cases of tuberculosis resistant to drug resistance. Shaanxi Medical Journal 38: 757-758.

39. Zhang Y, Li Q (2009) 84 patients with sputum smear-positive pulmonary tuberculosis drug resistance. Herald of Medicine 28: 1218-1219.

40. Tong Y, Wang Q (2009) The results of 89 cases sputum culture and drug susceptibility analysis. Chinese Journal of Primary Medicine And Pharmacy 16: 641-642.

41. Wu Y, Li H (2010) Analysis of tuberculosis drug-resistance of isoniazid with lectra lung disease. Journal of Clinical Pulmonary Medicine 15: 699-700.

42. Zhang Y, Xiong M, Cai C, Luo K, Wang Y, et al. (2010) Clinical Analysis of Extensive Drug Resistant Tuberculosis. Journal of Clinical Pulmonary Medicine 15: 1130-1131.

43. Shi X, Chen Q, Bai S, Zhang W (2010) Investigation of mycobacterium tuberculosis resistance in Nanjing. Chinese Journal of Clinical Laboratory Science 28: 234-235.

44. Duan H (2010) Drug resistance in tuberculosis sputum: a report of 568 case. Journal of Shanxi Medical University 41: 634-636.

45. Chen J, Yang G, Luo M (2010) Studies on drug resistance and drug resistant spectrum of mycobacterium Tuberculosis in sichuan area. Modern Preventive Medicine 37: 3211-3212.

46. Wu G, Zhou X, Luo T, Mao Y, Ma Y, et al. (2010) Analysis of the anti-tuberculosis drugs resistance of mycobacterium tuberculosis In chengdu area. Modern Preventive Medicine 37: 1753-1754.

47. Fan Y, Zhou Z, Yang S, Li H, Gao L, et al. (2010) Etiological diagnosis and drug resistance of HIV/ AIDS coinfected with tuberculosis in Yunnan province. Chinese Journal of Aids & STD: 359-363.

48. Zeng D, Lin S (2010) Survey of resistance of 713 Mycobacterium tuberculosis strains to antibiotics. China Tropical Medicine 10: 688, 702.

49. Li Q, Zhang C, Yan X (2010) Analysis of the Bacterial Type and Drug Resistance of 443 Tuberculosis Mycobacterium Strains. Chinese Journal of Medcinal Guide: 352, 354.

50. Hong J, Jin X, Guo X (2010) Monitoring of new registration TB drug resistance cases in songjiang area of Shanghai nearly eight years. Chinese Journal of Disease Control & Prevention: 819-820.

51. Wu Z, Zhang Z, Xu J (2010) Preliminary epidemiological study of drug-resistant spinal tuberculosis in Chongqing. Chongqing Medical Journal 39: 1420-1421.

52. Zhang X, Hu C, Wang S, Li M (2010) Analysis of drug resistance spectrums of 194 cases of resistant tuberculosis. Chinese Journal of Tuberculosis And Respiratory Diseases 33: 145-146.

**Review:**

1. Tan H (2004) Comparison of four epidemiological survey of tuberculosis bacterial resistance in Hunan Province. Practical Preventive Medicine 11: 119-120.

**Non-standard DST:**

1. Feng Q, Fan S, Wei J, Gao A (1994) An analysis of drug- resistance of 243 patients with refractory pulmonary tuberculosis. Journal of Xinxiang Medical College 4: 299-301.

2. Zhang C, He G, Huang Y (2003) Trend of TB Drug Resistance in Kaifeng City. Bulletin of Chinese Antituberculosis Association 25: 276-277.

3. Meng M, Wu G (2007) Preliminary appraisal of bacterium type and analysis of drug resistance in tuberculosis patients' sputum culture in Haikou City. Guangdong Medical Journal 28: 1971-1972.

4. Dong M, Li Y, Kuang T, Sun B, Sun M, et al. (2009) A survey of the drug resistance in Clinical isolates of Mycobacterium tuberculosis and the epidemic trend of multidrug-resistant tuberculosis. Chinese Journal of Laboratory Diagnosis 13: 569-571.

5. Li Q, Wang X, Deng Y, Wang J (2010) Utilization of Antituberculosis Drugs and Drug Resistance in Our Hospital during the Period of 2006～2009. China Pharmacy: 3569-3571

**Data from Macao:**

1. Zhang S, Liu Y (2007) Epidemiological survey on the trend of drug resistance of Mycobacterium tuberculosis complex in Macao during 2001 to 2005. Chinese Journal of Tuberculosis and Respiratory Diseases 30: 411-414.

**Non-standard definition of Initial resistance**

1. Zhang C, Liu Y, Li Z (1994) Analysis of 100 cases tuberculosis patient resistance and treatment effect. Chinese Journal of Tuberculosis And Respiratory Diseases 5: 262.

**Data on *Atypical mycobacteria*:**

1. Xiao A, Wang W, Qin Z (2000) Analysis of 70 cases tuberculosis drug resistance. Journal of Internal Intensive Medicine 6: 68-70.

2. Xiao X, Wu J (2006) Analysis of 165 cases of resistant tuberculosis. Journal of Clinical Pulmonary Medicine 11: 230-231.

3. Yang H (2006) Analysis of resistant tuberculosis patients in Shijiazhuang. Chinese Journal of Health Laboratory Technology 16: 999-1000.

4. Zhang S, Mei L (2006) Detection of 375 cases mycobacterium tuberculosis initial drug resistance. Occupation and Health 22: 1276-1277.

5. Sun Z, Liu J, Lv C, Li X, Li Y (2007) Results of 157 cases of drug resistance of Mycobacterium in Shijiazhuang. Clinical Focus 22: 1655-1656.
