# Supplementary material for: Prevalence of Drug-Resistant Tuberculosis in Mainland China: Systematic Review and Meta-Analysis
Source: PLoS One. 2011 Jun 3;6(6):e20343. doi: 10.1371/journal.pone.0020343 (PMC3108589; doi:10.1371/journal.pone.0020343)
Supplement: Table S3 — Distribution of different patterns of TB drug resistance among new cases in China. (DOC) [file pone.0020343.s007.doc]

**Table S3. Distribution of different patterns** of TB drug resistance among new cases in China

|  | **Mono-drug resistance /n**  **(95% CI)** | | | | **Multi-drug resistance/n**  **(95% CI)** | | | | **Resistance to specific drug * /n**  **(95% CI)** | | | |
| --- | --- | --- | --- | --- | --- | --- | --- | --- | --- | --- | --- | --- |
|  | **H** | **R** | **S** | **E** | **HR** | **HRS** | **HRE** | **HRSE** | **H** | **R** | **S** | **E** |
| **Total** | 4.1/38  (3.5-4.8) | 1.8/37  (1.2-2.3) | 4.8/38  (3.9-5.9) | 0.8/37  (0.5-1.3) | 1.6/45  (1.2-2.0) | 2.0/44  (1.5-2.5) | 0.6/40  (0.4-0.8) | 1.7/46  (1.2-2.2) | 15.3/71  (13.1-17.7) | 8.9/71  (7.5-10.6) | 14.3/71  (12.4-16.5) | 4.9/71  (3.6-6.1) |
| **Stratified by geographic areas** | | | | | | | | | | | | |
| North China | 2.6/6  (1.5-4.5) | 1.3 /5  (0.6-2.6) | 3.6/6  (2.2-5.6) | 1.4/5  (0.5-3.8) | 1.5/5  (0.9-2.4) | 1.2/6  (0.6-2.4) | 1.0/6  (0.4-2.5) | 2.0/6  (1.1-3.5) | 14.8/11  (10.3-20.8) | 8.5/11  (5.8-12.4) | 15.1/11  (11.0-20.4) | 5.7/11  (3.3-9.7) |
| East China | 3.5/13  (2.6-4.7) | 1.8/13  (1.2-2.7) | 5.0/13  (3.8-6.6) | 0.6/13  (0.2-1.5) | 1.1/18  (0.8-1.4) | 1.5/18  (1.1-2.0) | 0.5/17  (0.3-0.8) | 1.1/17  (0.7-1.6) | 13.8/30  (10.2-18.3) | 6.9/30  (5.2-9.2) | 12.9/30  (10.1-16.3) | 3.8/30  (2.6-5.6) |
| South China | 5.5/8  (4.4-7.0) | 1.9/8  (1.0-3.6) | 4.3/8  (2.0-8.8) | 0.9/8  (0.5-1.6) | 1.8/8  (0.9-3.4) | 1.5/7  (1.1-2.2) | 0.9/7  (0.5-1.7) | 1.4/8  (1.1-1.7) | 11.4/10  (12.1-16.4) | 8.2/10  (6.4-10.4) | 9.7/10  (6.6-14.1) | 5.2/10  (3.4-7.8) |
| Central China | 4.4/9  (3.4-5.8) | 2.0/9  (1.0-4.3) | 5.8/9  (4.8-6.9) | 0.7/9  (0.3-1.5) | 2.6/8  (2.1-3.3) | 5.5/8  (3.8-7.9) | 0.5/7  (0.3-0.9) | 6.0/8  (4.6-7.7) | 22.9/12  (18.0-28.7) | 15.9/12  (12.3-20.2) | 22.7/12  (18.0-28.2) | 9.3/12  (5.5-12.2) |
| West China | 5.8/2  (4.5-7.6) | 1.4/2  (0.8-2.2) | 7.2/2  (2.8-17.3) | 1.3/2  (0.6-2.9) | 2.4/6  (1.2-4.9) | 3.9/5  (2.5-6.1) | 0.5/4  (0.3-0.8) | 1.7/7  (0.7-4.2) | 13.7/8  (7.9-22.7) | 11.1/8  (5.3-22.0) | 16.0/8  (9.8-25.0) | 4.2/8  (1.5-11.2) |
| **Stratified by years** | | | | | | | | | | | | |
| Before (include) 2000 | 3.7/21  (2.9-4.7) | 1.2/20  (0.8-1.8) | 4.3/21  (3.5-5.4) | 0.5/20  (0.2-1.1) | 1.5/24  (1.1-2.1) | 1.9/23  (1.2-2.9) | 0.4/19  (0.2-0.8) | 1.8/23  (1.2-2.6) | 15.1/33  (12.3-18.2) | 7.6/33  (6.0-9.5) | 12.6/33  (10.0-15.9) | 4.0/33  (2.9-5.5) |
| After 2000 | 5.1/17  (4.2-6.3) | 2.2/17  (1.4-3.3) | 4.5/17  (2.9-6.7) | 1.1/17  (0.7-1.6) | 1.7/21  (1.2-2.3) | 1.9/21  (1.4-2.6) | 0.6/20  (0.4-1.0) | 1.5/21  (1.1-2.1) | 14.5/33  (11.6-18.1) | 9.3/33  (7.3-11.8) | 14.2/33  (11.6-17.3) | 4.6/33  (3.3-6.5) |
| **Stratified by DST methods** | | | | | | | | | | | | |
| Absolute concentration method | 3.9/19  (2.9-5.1) | 2.118  (1.4-3.0) | 4.4/19  (3.0-6.4) | 1.1/18  (0.6-2.1) | 1.4/23  (0.9-2.3) | 1.7/23  (1.1-2.5) | 0.5/19  (0.3-1.0) | 1.2/25  (0.7-2.2) | 15.1/35  (11.6-19.4) | 9.3/34  (7.0-12.3) | 14.6/34  (11.4-18.5) | 5.3/34  (3.8-7.4) |
| The proportion method | 4.1/17  (3.3-5.0) | 1.8/17  (1.2-2.5) | 5.5/17  (4.5-6.7) | 0.7/17  (0.4-1.3) | 1.5/19  (1.3-1.8) | 2.3/19  (1.6-3.2) | 0.5/19  (0.3-0.9) | 2.1/19  (1.5-2.9) | 14.8/29  (12.5-17.4) | 9.1/29  (7.5-11.0) | 16.0/29  (13.6-18.6) | 4.6/29  (3.4-6.1) |
| BACTEC | 4.1/2  (2.5-6.8) | 0.6/2  (0.3-0.9) | 2.9/2  (1.1-7.6) | 0.1/2  (0.0-0.4) | 2.3/3  (1.7-3.2) | 1.8/3  (0.9-3.6) | 1.7/3  (0.8-3.5) | 1.8/3  (0.6-5.2) | 15.7/7  (9.6-24.8) | 7.4/7  (5.2-10.4) | 8.7/7  (6.4-11.6) | 5.9/7  (2.9-11.6) |

Abbreviation: BACTEC, BACTEC 460 TB system of anti-tubercular screening; DST, drug-susceptibility testing; E, ethambutol; H, isoniazid; R, rifampicin; S, streptomycin.

* Resistance to specific drug regardless of mono drug resistance or multiple drug resistance.
