# Supplementary material for: Prevalence of Drug-Resistant Tuberculosis in Mainland China: Systematic Review and Meta-Analysis
Source: PLoS One. 2011 Jun 3;6(6):e20343. doi: 10.1371/journal.pone.0020343 (PMC3108589; doi:10.1371/journal.pone.0020343)
Supplement: Table S4 — Distribution of different patterns of TB drug resistance among previously treated cases in China. (DOC) [file pone.0020343.s008.doc]

**Table S4. Distribution of different patterns** of TB drug resistance among previously treated cases in China

|  | **Mono-drug resistance /n**  **(95% CI)** | | | | **Multi-drug resistance/n**  **(95% CI)** | | | | **Resistance to specific drug * /n**  **(95% CI)** | | | |
| --- | --- | --- | --- | --- | --- | --- | --- | --- | --- | --- | --- | --- |
|  | **H** | **R** | **S** | **E** | **HR** | **HRS** | **HRE** | **HRSE** | **H** | **R** | **S** | **E** |
| **Total** | 6.2/25  (4.3-8.8) | 2.7/25  (1.5-4.6) | 5.4/25  (3.8-7.6) | 1.4/25  (0.9-2.1) | 7.1/36  (5.6-8.8) | 8.7/33  (6.7-11.2) | 3.6/33  (2.9-4.4) | 9.1/35  (7.0-11.7) | 41.5/50  (36.6-46.6) | 34.6/50  (30.1-39.4) | 32.9/50  (28.9-37.2) | 18.1/50  (15.3-21.2) |
| **Stratified by geographic areas** | | | | | | | | | | | | |
| North China | 2.3/3  (1.0-5.1) | 1.7/3  (0.8-3.6) | 3.4/3  (1.2-8.7) | 2.7/2  (0.9-7.8) | 4.2/3  (2.8-6.3) | 5.2/3  (4.6-5.9) | 5.7/3  (2.8-11.4) | 3.8/3  (2.5-5.9) | 46.5/9  (38.5-54.7) | 37.3/9  (31.4-43.6) | 34.6/9  (29.6-40.0) | 25.7/9  (19.9-32.5) |
| East China | 9.2/9  (5.2-15.7) | 3.0/9  (0.9-9.4) | 5.4/9  (2.6-10.8) | 1.0/9  (0.3-3.0) | 6.7/17  (4.9-9.1) | 9.3/16  (7.6-11.3) | 3.8/16  (2.7-5.1) | 11.4/17  (8.8-14.7) | 44.1/21  (35.6-52.9) | 35.9/21  (28.1-44.6) | 34.1/21  (28.1-40.6) | 19.9/21  (15.4-25.4) |
| South China | 8.8/6  (6.4-12.1) | 3.0/6  (1.8-5.0) | 7.0/6  (2.6-17.5) | 1.6/6  (0.7-3.8) | 6.8/7  (4.5-10.2) | 4.0/6  (2.3-6.9) | 4.1/6  (2.9-5.7) | 6.9/7  (4.4-10.8) | 35.7/8  (28.4-43.7) | 27.4/8  (20.1-36.1) | 25.3/8  (18.3-33.9) | 13.5/8  (9.9-18.2) |
| Central China | 4.4/5  (3.4-5.6) | 2.6/5  (1.8-3.8) | 6.0/5  (4.2-8.5) | 0.6/5  (0.3-1.2) | 8.0/5  (1.9-27.8) | 12.6/4  (8.6-18.0) | 1.8/4  (1.2-2.8) | 14.0/4  (8.4-22.5) | 43.4/7  (39.4-47.6) | 38.8/7  (32.9-45.1) | 40.0/7  (32.6-47.7) | 20.5/7  (16.4-25.3) |
| West China | 3.3/2  (1.7-6.3) | 2.8/2  (1.4-5.6) | 2.8/2  (0.6-11.3) | 0.4/2  (0.1-2.5) | 8.4/4  (4.6-14.8) | 21.7/4  (11.7-36.6) | 2.4/4  (1.1-5.2) | 9.1/4  (4.3-18.3) | 30.4/5  (8.0-68.7) | 32.6/5  (9.0-70.2) | 28.9/5  (8.2-65.0) | 7.2/5  (2.2-21.1) |
| **Stratified by years** | | | | | | | | | | | | |
| Before (include) 2000 | 5.5/10  (3.8-7.9) | 2.2/10  (1.2-4.0) | 5.2/10  (3.6-7.5) | 0.6/10  (0.4-1.0) | 7.1/13  (4.2-11.6) | 8.7/11  (5.8-12.8) | 2.8/11  (1.8-4.4) | 9.7/12  (6.5-14.2) | 41.6/16  (33.8-49.8) | 34.9/16  (26.9-43.8) | 30.5/16  (23.1-39.1) | 17.9/16  (13.3-23.7) |
| After 2000 | 7.6/13  (4.8-11.8) | 3.3/13  (1.5-7.1) | 5.0/13  (2.5-10.0) | 1.9/13  (1.1-3.3) | 6.9/20  (5.2-9.2) | 8.0/19  (5.2-12.1) | 3.5/19  (2.6-4.7) | 7.5/20  (5.4-10.2) | 38.7/28 (33.4-44.3) | 32.4/28  (26.6-38.8) | 32.4/28  (27.7-37.5) | 16.0/28  (12.5-20.1) |
| **Stratified by DST methods** | | | | | | | | | | | | |
| Absolute concentration method | 7.0/10  (3.7-12.8) | 4.5/10  (1.9-10.3) | 4.7/10  (1.9-11.2) | 2.9/10  (1.7-4.8) | 7.6/15  (4.9-11.7) | 9.3/13  (6.0-14.1) | 3.3/13  (2.3-4.8) | 6.9/14  (4.6-10.2) | 47.6/18  (40.6-54.7) | 42.5/18  (34.8-50.6) | 39.9/18  (33.8-46.3) | 21.5/18  (17.2-26.5) |
| The proportion method | 4.9/12  (3.0-7.7) | 2.2/12  (1.5-3.3) | 6.4/12  (5.3-7.7) | 0.6/12  (0.2-1.7) | 6.4/16  (4.7-8.6) | 8.6/16  (5.8-12.5) | 3.2/16  (2.4-4.3) | 9.2/16  (6.1-13.6) | 38.3/25  (34.5-42.3) | 32.2/25  (28.7-35.8) | 30.2/25  (26.6-34.1) | 17.2/25  (14.3-20.5) |
| BACTEC | 10.2/3  (8.7-11.9) | 1.4/3  (0.9-2.2) | 4.2/3  (2.0-8.8) | 0.6/3  (0.2-2.5) | 7.8/5  (3.7-15.6) | 7.2/4  (3.0-16.5) | 5.7/4  (3.1-10.0) | 16.6/5  (9.4-27.5) | 43.7/6  (17.5-74.0) | 30.6/6  (12.5-57.6) | 29.0/6  (12.2-54.6) | 19.5/6  (7.4-42.5) |

Abbreviation: BACTEC, BACTEC 460 TB system of anti-tubercular screening; DST, drug-susceptibility testing; E, ethambutol; H, isoniazid; R, rifampicin; S, streptomycin.

* Resistance to specific drug regardless of mono-drug resistance or multi-drug resistance.
